# Elucidating the Hydrogen Selectivity of Pt/TiO_*x*_/C as a Fuel-Cell Catalyst by Operando Near-Ambient-Pressure XPS

**DOI:** 10.1021/acsami.5c09047

**Published:** 2025-08-30

**Authors:** Nhat Long Tran Pham, Simon Qian, Thomas Götsch, Corbinian Grön, Juan Jesus Velasco-Vélez, Björn M. Stühmeier, Axel Knop-Gericke, Hubert A. Gasteiger, Michele Piana

**Affiliations:** † 9184 Technical University of Munich, TUM School of Natural Sciences, Department of Chemistry and Catalysis Research Center, Chair of Technical Electrochemistry, Lichtenbergstr. 4, 85748 Garching b. München, Germany; ‡ 28259 Fritz-Haber-Institut der Max-Planck-Gesellschaft, Department of Inorganic Chemistry, Faradayweg 4-6, 14195 Berlin, Germany; § Max Planck Institute for Chemical Energy Conversion, Stiftstraße 34-36, 45470 Mülheim an der Ruhr, Germany

**Keywords:** strong metal−support interaction, Pt/C, Pt/TiO_*x*_/C, fuel cell, catalysts, operando, near-ambient-pressure, X-ray photoelectron spectroscopy

## Abstract

The long-term stability of proton exchange membrane fuel cells (PEMFCs) faces significant challenges, particularly during start-up and shut-down events, which lead to degradation of the cathode catalyst through the oxidation of its carbon support. To improve catalyst durability, an anode catalyst with a high selectivity toward the hydrogen oxidation/evolution reaction rather than the oxygen reduction reaction is necessary. Pt/TiO_*x*_/C (*x* < 2) catalysts have been reported to provide excellent hydrogen selectivity due to its strong metal–support interaction (SMSI) between Pt particles and TiO_*x*_ support. To further elucidate the SMSI-induced effect of the catalyst, this study employs near-ambient-pressure X-ray photoelectron spectroscopy (NAP-XPS) at BESSY II with an upgraded operando cell, optimized for the use of membrane electrode assemblies (MEAs) for the first time. The electrochemical behavior of the operando cell is fully consistent with PEMFC measurements for both the standard Pt/C and investigated Pt/TiO_*x*_/C catalysts. With NAP-XPS, the SMSI-induced effect is observed through a significant suppression of Pt oxidation at high potentials for Pt/TiO_*x*_/C. A precise quantification of the oxidation charge from both electrochemical and NAP-XPS data evidently shows partial Pt oxidation for Pt/TiO_*x*_/C, clearly originating from Pt deposited on carbon instead of TiO_*x*_, as demonstrated by transmission electron microscopy. Nevertheless, the results reveal that barely any oxidation is expected for SMSI-based catalysts such as pure Pt/TiO_*x*_/C. Cracks in the bilayer graphene used as an X-ray transparent window in the operando setup likely explain the lower absolute values in Pt oxidation obtained from NAP-XPS compared with the values from electrochemistry, still allowing valuable insights into the catalyst behavior.

## Graphical abstract

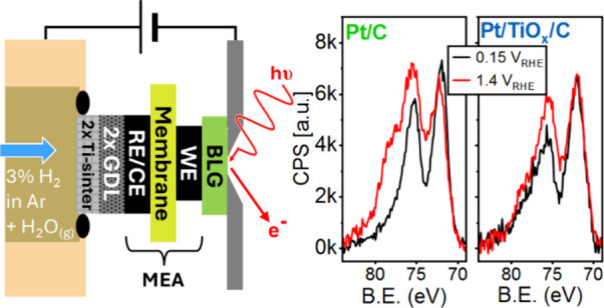

## 1 Introduction

The long-term stability of proton exchange membrane fuel cells (PEMFCs) is notably affected by the cathode degradation, especially during start-up and shut-down (SUSD) events, as was first illustrated by Reiser et al. and is continuously investigated.^1^ During these transient conditions, a hydrogen-air gas front moves through the anode flow field, driving the oxygen reduction reaction (ORR) in the air-filled segment. Simultaneously, at the opposite cathode side, those events trigger carbon and water oxidation, leading to accelerated catalyst degradation and reduced performance. To prevent SUSD events on a catalyst level, i.e., without relying on system control solutions, an anode catalyst with a high selectivity toward the hydrogen evolution reaction/hydrogen oxidation reaction (HOR/HER) and, thus, low ORR activity is desired.^2^ A Pt/TiO_*x*_/C (with *x* < 2) catalyst is a promising candidate to pursue this strategy.^2,3^ This type of catalyst was synthesized by Stühmeier et al., who showed with the rotating-disk-electrode (RDE) technique, that a proper level of HOR/HER activity (≈30% vs Pt/C in alkaline) can be maintained with a strongly suppressed ORR activity (≈2% vs Pt/C in acidic electrolyte).^3^ Noticeably, the catalyst is also able to sustain a high HOR/HER activity even at potentials of ≥1.0 V_RHE_ (RHE ≡ reversible hydrogen electrode), where typically deactivation occurs due to Pt-oxide formation. The origin of these observations is ascribed to a reductive heat-treatment on the Pt/TiO_*x*_/C catalyst, which is reported to produce a strong metal–support interaction (SMSI) induced by partially reduced TiO_*x*_ layer encapsulating the Pt particles.^3,4^ Generally, the SMSI effect between a metal and a suitable oxide support is known and was applied to various heterogeneous catalytic reactions.^5–7^ In the particular case of Pt/TiO_*x*_/C, the TiO_*x*_ layer on the Pt particle is hypothesized to be permeable to protons but impermeable to any oxygen-containing species, explaining the observed electrochemical behavior at high potential in rotating-disk-electrode (RDE) experiments.^3,4,8^ The catalyst was further tested in single cell PEMFC tests for its SUSD durability, where a beneficial effect toward preventing SUSD degradation was also observed.^9^

To further demonstrate and understand the origin behind the enhanced selectivity of Pt/TiO_*x*_/C, traditional ex situ characterization methods, although valuable for understanding catalyst structure and composition, fall short in capturing the dynamic processes occurring during fuel cell operation. This limitation has been overcome in plenty of electrocatalysis studies with the adoption of operando techniques, also using near-ambient-pressure XPS (NAP-XPS),^10,11^ which enable monitoring of catalysts under controlled conditions in an electrochemical cell. Several attempts to achieve such an electrochemical operando cell setup have already been reported. Former cell designs were rather simple, using a membrane-electrode assembly (MEA), produced by hot-pressing electrodes onto both sides of an ionomeric membrane, and applied in studies about the potential-dependent oxidation of either Pt or bimetallic PtAu (both unsupported) in high-temperature PEMFCs; unfortunately, the electrochemical results collected with this cell and the choice of the reference electrode (in situ dynamic hydrogen electrode) were not optimal.^12–15^ Furthermore, operando electrochemical cells have also been employed to investigate catalysts for electrolysis (in particular Ir-based), with an iterative optimization of the cell over several years by various authors.^11,16–19^ This kind of operando cell usually involved the use of an aqueous electrolyte to humidify the backside of the MEA, while the electrodes were usually sputtered onto the proton exchange membrane (PEM) and the investigated electrode was facing toward the analysis chamber. An Ag/AgCl reference electrode was immersed in the liquid electrolyte at most cell iterations in order to properly determine the potential and enable better-controlled electrochemical analysis. With this cell design for NAP-XPS, Mom et al. studied Pt oxidation in a PEMFC-like environment (sputtered Pt on Nafion 117) upon increasing the electrode potential. They suggested the formation of a mixture of Pt surface oxides (Pt^δ+^, Pt^2+^, and Pt^4+^) above the Pt-oxide onset potential of ≈0.9 V_RHE_, while even higher potentials increased the amount of Pt^4+^ bulk oxides.^20^ The electrochemical analysis was not optimal due to the high electrical resistance of 50 Ω for their cell setup and an unsatisfactory Pt utilization due to the use of a sputtered Pt electrode (up to 16 nm thick) directly deposited on the membrane.^11,20^ The research literature still misses NAP-XPS studies employing MEAs similar to those used in PEMFCs, with the aim of reproducing their electrochemistry during voltammetric measurements. Furthermore, a NAP-XPS study on Pt/TiO_*x*_/C, to determine and confirm the origin behind its enhanced selectivity, is also lacking.

In this work, we present further optimization of the cell design in the literature, reporting results obtained with an improved NAP-XPS operando cell setup, adapted from the design by Velasco-Vélez et al., for use at the ISISS workstation at BESSY II.^11^ This setup is specifically tailored to incorporate a MEA that closely replicates those used in standard PEMFCs. This includes the absence of any liquid aqueous electrolytes and the use of catalyst layers instead of sputtered catalysts, enabling a more accurate assessment of catalyst behavior under conditions closer to actual application. Furthermore, the same operando cell allows simultaneous quantification of Pt-oxide formation from electrochemical and NAP-XPS data and their direct correlation. By comparing the Pt/TiO_*x*_/C with a standard Pt/C catalyst, we aim to elucidate the role of the SMSI-induced TiO_*x*_ layer, its impact on Pt oxidation, and, in general, the electrochemical behavior of such catalyst.

## 2 Experimental Section

### 2.1 Membrane-Electrode Assembly (MEA) Preparation

The working electrodes (WEs) for this study used two different catalyst powders, i.e., Pt/C (19.8 wt % Pt, TEC10V20E, Tanaka Kikinzoku Kogyo, TKK, Japan) and a synthesized, heat-treated Pt/TiO_*x*_/C (13.0 wt % Pt, 21.6 wt % Ti, 41 wt % C), both with an average Pt particle size diameter of ≈3 nm.^9^ The synthesis of the Pt/TiO_*x*_/C catalyst is described in detail elsewhere.^3,9^ It is further noted that the Pt/TiO_*x*_/C catalyst powder was not used as-synthesized, and an additional heat treatment, according to Stühmeier et al., was performed to produce an SMSI-induced TiO_*x*_ encapsulation of the Pt particle, enabling its unique electrochemistry.^3,9^ The MEA preparation was carried out using the decal transfer method by hot-pressing the same electrodes used to collect the data reported by Stühmeier et al. on the PEM.^9^ The procedures for manufacturing the electrode ink and decals are described elsewhere,^9,21^ but the most pertinent information is given in the following.

The counter/reference electrode (CE/RE) of the MEA was made with a Pt/C catalyst with a higher Pt-content (45.6 wt % Pt, TEC10V50E, TKK, Japan). Catalyst inks were prepared as follows. For Pt/C-based inks, 0.02 g_support_ mL_ink_^–1^, an ionomer/support (I/S) ratio of 0.65, and a water content of 7.5 wt % were used, while for Pt/TiO_*x*_/C-based inks, 0.012 g_support_ mL_ink_^–1^, an I/S of 0.55, and a water content of 2.5 wt % were used. The catalyst powder (≈0.187 g for 20 wt % Pt/C or ≈0.069 g for Pt/TiO_*x*_/C) was added into an 8 mL HDPE bottle (for ≈5 mL ink), along with 16.5 g of 5 mm ZrO_2_ beads (very high density ZrO_2_, Glen Mills Inc., USA), 1-propanol (≈3.66 g for 20 wt % Pt/C or ≈3.90 g for Pt/TiO_*x*_/C, ≥99.9%, Sigma-Aldrich, Germany) as a solvent, and an ionomer dispersion (0.40 g for 20 wt % Pt/C or 0.13 g for Pt/TiO_*x*_/C, ≈25 wt % of a 700 equiv weight in water, Asahi Kasei, Japan). The components were mixed for 18 h on a roller-mill (BTR-12 V, Ratek, Australia) at 60 rpm and room temperature and afterward coated onto a PTFE sheet (virgin PTFE, 50 μm, Angst+Pfister, Germany) with the Mayer-rod technique (coating machine: K control coater; RK PrintCoat Instruments, Ltd., England). For the coating, an appropriate rod size (wet film thickness of 70 μm for 20 wt % Pt/C and ≈80 μm for Pt/TiO_*x*_/C) was chosen to achieve the following electrode loadings: 45 ± 5 μg_Pt_ cm_MEA_^–2^ for the WE and 300 ± 25 μg_Pt_ cm_MEA_^–2^ for the CE/RE. The air-dried electrode decals were then cut into circles with a diameter of 3 mm for the WE and 6 mm for the CE/RE and then hot-pressed onto a Nafion 212 membrane (50 μm, Quintech, Germany) at 155 °C for 3 min under a pressure of 0.11 kN cm^–2^. The larger size of the CE/RE was chosen to ensure that this electrode area was not limiting and thus affecting the WE electrochemistry. The actual electrode loadings were determined by weighing the decals before (electrode + PTFE) and after (only PTFE) the hot-pressing procedure.

### 2.2 Operando Cell Setup

The operando cell setup was adapted from a cell designed by Velasco-Veléz et al. and optimized for measurements with an MEA in order to resemble a conventional PEMFC.^11,18,20,22–25^ Figure 1 illustrates a schematic view and a detailed exploded view of the operando cell design. Prior to the assembly of the operando cell, a bilayer graphene (BLG, Graphenea, Spain) sheet was prepared and placed on top of the WE, as described in detail in the literature.^20,22^ The BLG acts as an X-ray and photoelectron transparent window (400–600 eV kinetic energy) and serves as an evaporation barrier to maintain humidity in the topmost catalyst layer, allowing the oxidative reactant to be available at the Pt nanoparticles under near-ambient-pressure conditions, as mentioned by Mom et al.^11,20,22^ In the current modification of the cell design, a gas stream containing 3% H_2_ in Ar (mixture of hydrogen 3.0 and argon 4.6 (“Argon W3” gas), Westfalen AG, Germany), delivered through a water-filled gas bubbler, provides the humidified reactant to the CE/RE side of the cell. This hydrogen source is crucial for the CE/RE to function as a reference electrode. This reference potential corresponds to the reversible hydrogen electrode (RHE) potential at 25 °C and 30 mbar of H_2_ (3% H_2_ in Ar at ambient pressure), but all potentials reported in this work are referenced to the RHE potential at a nominal H_2_ pressure of 100 kPa_abs_ (defined as V_RHE_) by taking the Nernst shift of 49 mV into account.

**Figure 1:**
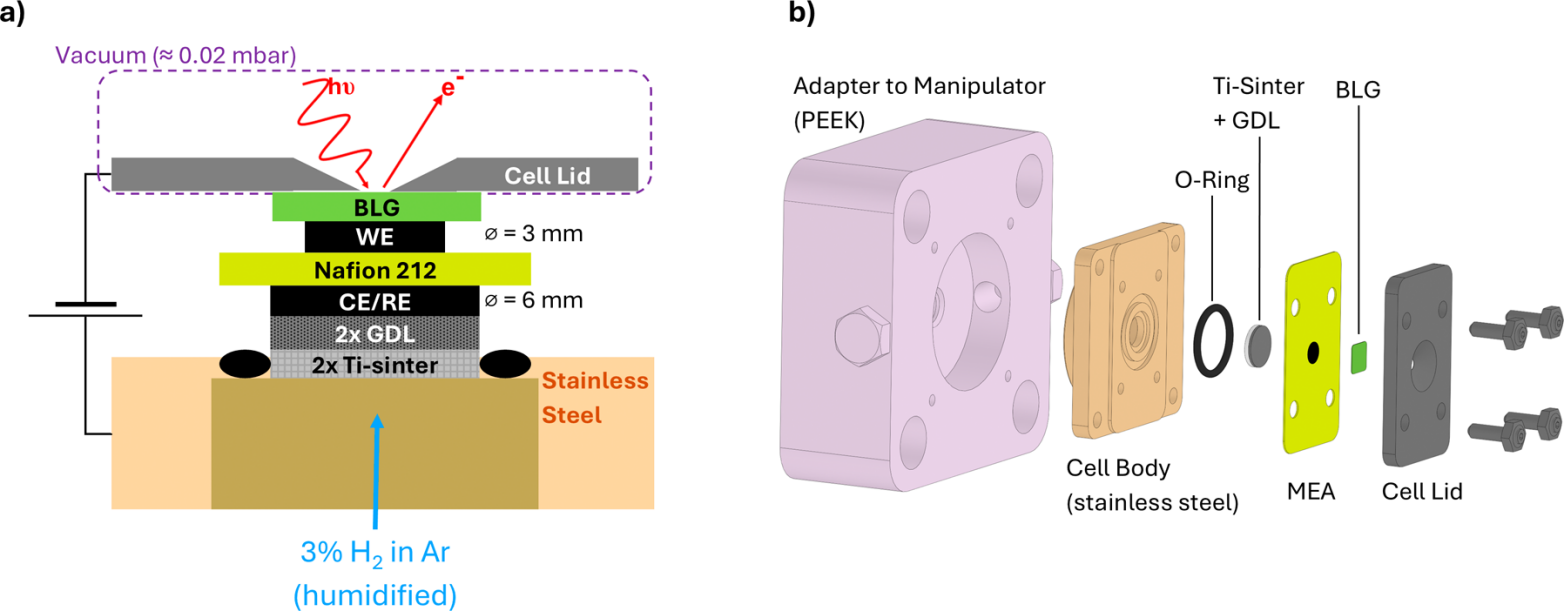
(a) Schematic illustration of the operando cell design employing a membrane-electrode assembly (MEA). The cell includes a porous Ti-sinter and gas diffusion layers (GDLs) that provide mechanical support and ensure proper compression of the MEA. The counter electrode (CE) also works as a reference electrode (RE) by supplying it with a 3% H_2_ in Ar gas mixture to establish a constant potential, as in a reversible hydrogen electrode (RHE). A bilayer graphene (BLG, green) is positioned on top of the working electrode (WE) to act as both an X-ray window and an evaporation barrier, maintaining humidity in the topmost catalyst layer. (b) Exploded view of the operando cell, adapted from the cell design by Velasco-Veléz et al.^11,18,20^

Additional components include two layers of a porous Ti-sinter (Series 1100, each ≈250–300 μm thick, Mott Corporation, USA) and two gas diffusion layers (GDLs, each ≈175 μm thick, H14C10, Freudenberg, Germany), positioned adjacent to the CE/RE of the MEA. These layers provide mechanical support, ensuring proper compression and contact between components while homogeneously distributing the humidified gas across the CE/RE area. The cell is sealed by hand-tightening a lid made of boron-doped diamond-coated niobium (with an open window having a diameter of 1.5 mm) onto FFPM O-rings (Dichtelemente Arcus GmbH, Germany), then attached to the manipulator at the ISISS beamline of the BESSY II/HZB synchrotron facility (Berlin, Germany). Electrical connections were established between the cell lid and the stainless-steel cell body, with the Nafion membrane also serving as an electrical insulator between the current collectors of WE and CE/RE. Electrochemical data are recorded using an SP-300 potentiostat (Bio-Logic Science Instruments SAS, France). Regarding X-ray measurements at the beamline, the X-ray beam incidences at an angle of 55° to the sample, whereas the analyzer axis is oriented perpendicular to the surface. The storage ring operated in top-up mode and provided a ring current of 300 mA. The photons had to pass a 50 nm thick SiN window, which separated the main chamber from the UHV system of the beamline.

### 2.3 Operando Measurement Procedure

The operando cell was used at RT with a dynamic vacuum of ≈0.02 mbar inside the measurement chamber. To check the overall consistency of the cell assembly, electrochemical impedance spectroscopy (EIS) from 1 MHz to 100 Hz at the open circuit voltage (OCV, ≈0.1 V_RHE_) with an amplitude of 0.5 mV was measured, and a consistent high-frequency resistance (HFR) of ≈5 Ω across all cells was determined. The operando cell was then conditioned by cyclic voltammetry (CV) between 0.045 and 1.2 V_RHE_ for 50 cycles at a scan rate of 100 mV s^–1^ to clean the catalyst surface from impurities. Following this conditioning, the potential was ramped at a rate of 10 mV s^–1^ from OCV (∼0.1 V_RHE_) to 1.4 V_RHE_, a potential where Pt oxidation is expected, thereby verifying the effectiveness of the BLG deposition on the WE, similar to Mom et al. and shown in Figure S2.^20^ Every XPS spectrum was measured at a different pristine spot of the electrode, which was not exposed to X-rays prior (beam spot size of 300 × 100 μm (width × height)) to exclude beam damage effects as much as possible. Even though 1.15 V_RHE_ is enough for Pt oxidation to be detected, as observed in a similar setup with sputter-deposited Pt (1–5 nm up to 16 nm thickness) on Nafion 117 by Mom et al.,^20^ a higher potential of 1.4 V_RHE_ was selected for the BLG test to ensure sufficient Pt oxidation, since the oxidation overpotential is sensitive and shifts toward higher potentials with decreasing relative humidity (RH).^26,27^ Nevertheless, we could confirm comparable electrochemical behavior of the operando MEA to a 5 cm^2^ single PEMFC with an average RH < 40%, as shown in Figure S3. These electrochemical results show strong improvements in terms of a lower overpotential for Pt oxidation compared to those reported by Mom et al.,^20^ primarily due to the use of an MEA with an optimized electrode composition with respect to the catalyst (supported on a commonly used Vulcan carbon black) and the proton-conducting ionomer, as would be used in PEMFCs. After the potential was allowed to equilibrate back to the OCV, several CVs were recorded between 0.045 and 1.2 V_RHE_ with scan rates of 150, 100, 50, and 10 mV s^–1^ for three cycles each to determine the redox features of the catalysts and their electrochemical surface areas (ECSAs).

The operando experiment was then conducted, involving a linear sweep voltammetry (LSV) step at 10 mV s^–1^ and a chronoamperometric (CA) step at the respective constant potential. For the latter, various potentials of interest were probed, including the hydrogen underpotential deposition (H_UPD_) region at 0.15 V_RHE_, the double layer (DL) capacitance region at 0.45 V_RHE_, and the Pt-oxide region at 1.0, 1.2, and 1.4 V_RHE_, with XPS Pt 4f spectra being collected over ≈5 min after ≈2–5 min of potential equilibration. The current response during this operando experiment is illustrated in Figure 2 for an MEA with Pt/C as WE. At each constant-potential step, XPS spectra of the Pt 4f (86–66 eV binding energy, 10 scans, 0.1 eV step^–1^) and C 1s (298–278 eV binding energy, 1 scan, 0.1 eV step^–1^) regions were collected, using an excitation energy of 871 eV and a photon flux of 7.91 × 10^10^ photon s^–1^ to probe enough of the Pt particle through the several overlayers (BLG, ionomer, SMSI-induced TiO_*x*_ layer), while limiting beam-induced damage of the ionomer with higher excitation energies and photon flux (see Section 2.5 for further estimations). The XPS spectra collected to verify the effectiveness of the BLG deposition were measured with the same parameters. Two XPS measurements at 1.4 V_RHE_ were performed consecutively on different fresh electrode spots, with the last measurement labeled as EoT (End of Test) to account for the increased Pt oxidation with time during the CA step at constant potential.^28,29^ Additionally, spectra in the S 2p (180–157 eV binding energy, 4 scans, 0.1 eV step^–1^), O 1s (542–524 eV binding energy, 2 scans, 0.1 eV step^–1^), and F 1s (695–682 eV binding energy, 2 scans, 0.1 eV step^–1^) regions were recorded at 0.15 and 1.4 V_RHE_ (EoT), using an excitation energy of 871 eV and a photon flux of 7.91 × 10^10^ photon s^–1^. The data presented in this work are acquired from one Pt/C and two Pt/TiO_*x*_/C operando measurements.

**Figure 2:**
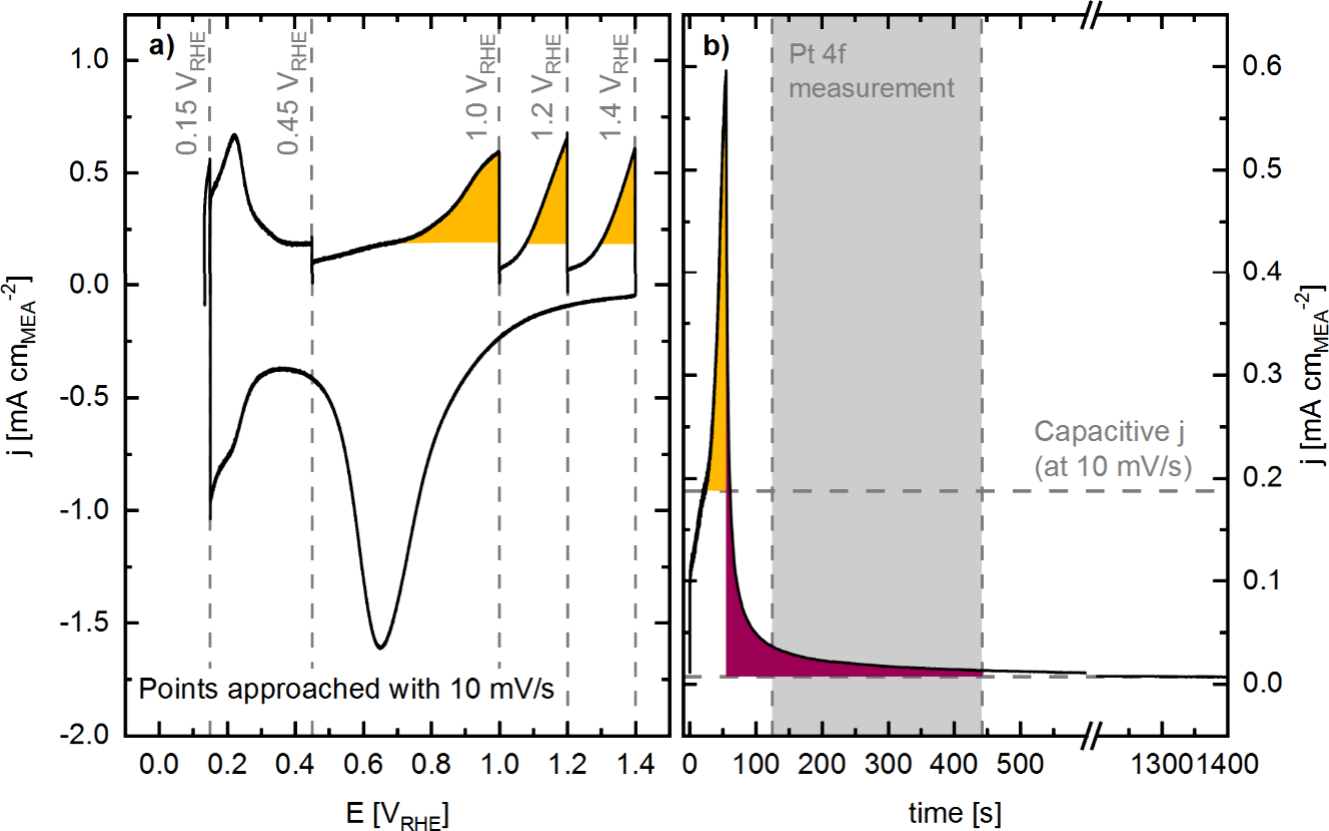
(a) Electrochemical data obtained from an MEA using Pt/C as the WE in the operando cell. Potentials were scanned at 10 mV s^–1^ and held constant during XPS spectra acquisition. The chosen potentials are indicated by light-gray dashed lines and correspond to the regions ascribed to hydrogen underpotential deposition (H_UPD_, 0.15 V_RHE_), double layer capacitance (0.45 V_RHE_), and Pt-oxide formation (1.0–1.4 V_RHE_). The yellow areas represent the charge associated with Pt-oxide formation during the positive-going scan at 10 mV s^–1^, whereby the lower integration limit corresponds to the capacitive current measured in the electrochemical double layer region at ≈0.45 V_RHE_ from CVs recorded prior to the operando measurement. (b) Exemplary current response of successive potentiodynamic (increasing current, yellow area) and potentiostatic (decreasing current, burgundy area) steps of the operando procedure from 0.45 to 1.0 V_RHE_. During the potentiodynamic step, the capacitive current density *j* is shown by the upper horizontal gray dashed line that limits the yellow area. The charge from the burgundy area corresponds to the Pt oxidation charge during the potentiostatic step. The time limit for the integral is defined by the end of the Pt 4f spectra acquisition, indicated by the gray area. The lower horizontal gray dashed line marks the lower current limit for the burgundy integral, set to the current measured 20 min after reaching the desired potential.

### 2.4 Electrochemical Quantification and Evaluation of Pt-Oxide Formation

To quantify the Pt-oxide formation for each catalyst, we calculated the associated electrons from both electrochemical and XPS data. In the electrochemical quantification of the LSV step of the operando experiment, the first portion of charge relative to Pt-oxide formation is represented by the yellow area shown in Figure 2a, with the lower current limit of the integral defined by the capacitive current at ∼0.45 V_RHE_ (*j*_cap_) determined from a CV prior to the operando experiment. The resulting charge of the LSV step is calculated as follows:

$$\int \left(j-j_{\mathrm{cap}}\right)dE\times \frac{A_{\mathrm{MEA}}}{\upsilon }=Q_{\mathrm{LSV}}$$ 1

Here, *j* is the current density during the LSV step, *E* is the potential, *A*_MEA_ is the area of the working electrode, υ is the scan rate, and *Q*_LSV_ is the resulting charge from the LSV step. Dividing this charge by the elementary charge yields the number of electrons *n*_e^–^,LSV_. Since Pt oxidation persists during the constant-potential chronoamperometric (CA) step, the charge during this step (indicated by the burgundy area in Figure 2b) also needs to be considered until the end of the Pt 4f measurement. The lower current density limit employed for the integration is set 20 min after the beginning of the CA step and corresponds to a background current density attributed to possible side reactions (≤1 μA cm_MEA_^–2^); such very small background current densities were consistently observed across all CA measurements. A possible higher faradaic current density from reactions such as oxygen reduction reaction (ORR) or oxygen evolution reaction (OER) was not observed. The resulting charge *Q*_CA_, which can be converted to the number of electrons during the CA step *n*_e^–^,CA_, is calculated by using the following equation:

2 $$\int jdt=Q_{\mathrm{CA}}$$

The total number of electrons associated with Pt oxidation results from the sum of the electrons from both steps, i.e., *n*_e^–^,LSV_ + *n*_e^–^,CA_. As the potential is incrementally increased (1.0 V_RHE_ → 1.2 V_RHE_ → 1.4 V_RHE_) without any negative-going potential excursions in between, the oxidation charges for each potential increment must be considered cumulatively in the analysis. To facilitate a comparison between electrochemical measurements and XPS quantification, the number of electrons is normalized to the number of Pt surface atoms Pt_sa_ of the Pt nanoparticles, calculated from the average Pt particle diameter of ≈3 nm as reported by Harzer et al. and Stühmeier et al. for Pt/C and Pt/TiO_*x*_/C catalysts, respectively, considering the average Pt surface density of 2.04 nmol_Pt_/cm^2^_Pt_ typically assumed for polycrystalline Pt.^3,9,21,30^ This normalization to electrons exchanged per Pt surface atoms, e^–^ Pt_sa_^–1^, is preferred over that to overall atoms since Pt oxidation directly relates to the surface of Pt particles rather than the bulk.

### 2.5 XPS Reference Sample Peak Model and Pt-Oxide Quantification

For representative references of the Pt^0^ oxidation state in the Pt/C and Pt/TiO_*x*_/C catalysts, operando samples at a potential of 0.15 V_RHE_ were used. Additionally, PtO_2_ powder (surface area ≥ 60 m^2^ g^–1^, Sigma-Aldrich, USA) was measured separately to obtain Pt^4+^ as well as Pt^2+^ oxidation state references (as explained below). The PtO_2_ powder was measured in its pristine form (i.e., without any pretreatment) at RT under ultrahigh vacuum (UHV, ≈10^–7^ mbar), and with an excitation energy of 471 eV and a photon flux of 7.56 × 10^9^ photon s^–1^. This excitation energy was chosen to be 400 eV lower than in the operando measurements to allow for a comparable XPS detection depth, accounting for the lack of the BLG window and of the ionomer overlayers in the reference sample as well as to minimize charging effects. Figure 3a–c shows the Pt 4f region of the measured reference samples (hollow black circles), namely, PtO_2_ powder for Pt^4+^ (but also for the Pt^2+^ species, as described below) as well as the operando measurements of the MEAs with Pt/C and Pt/TiO_*x*_/C working electrodes (WEs) held at 0.15 V_RHE_, serving as reference for Pt^0^.

**Figure 3:**
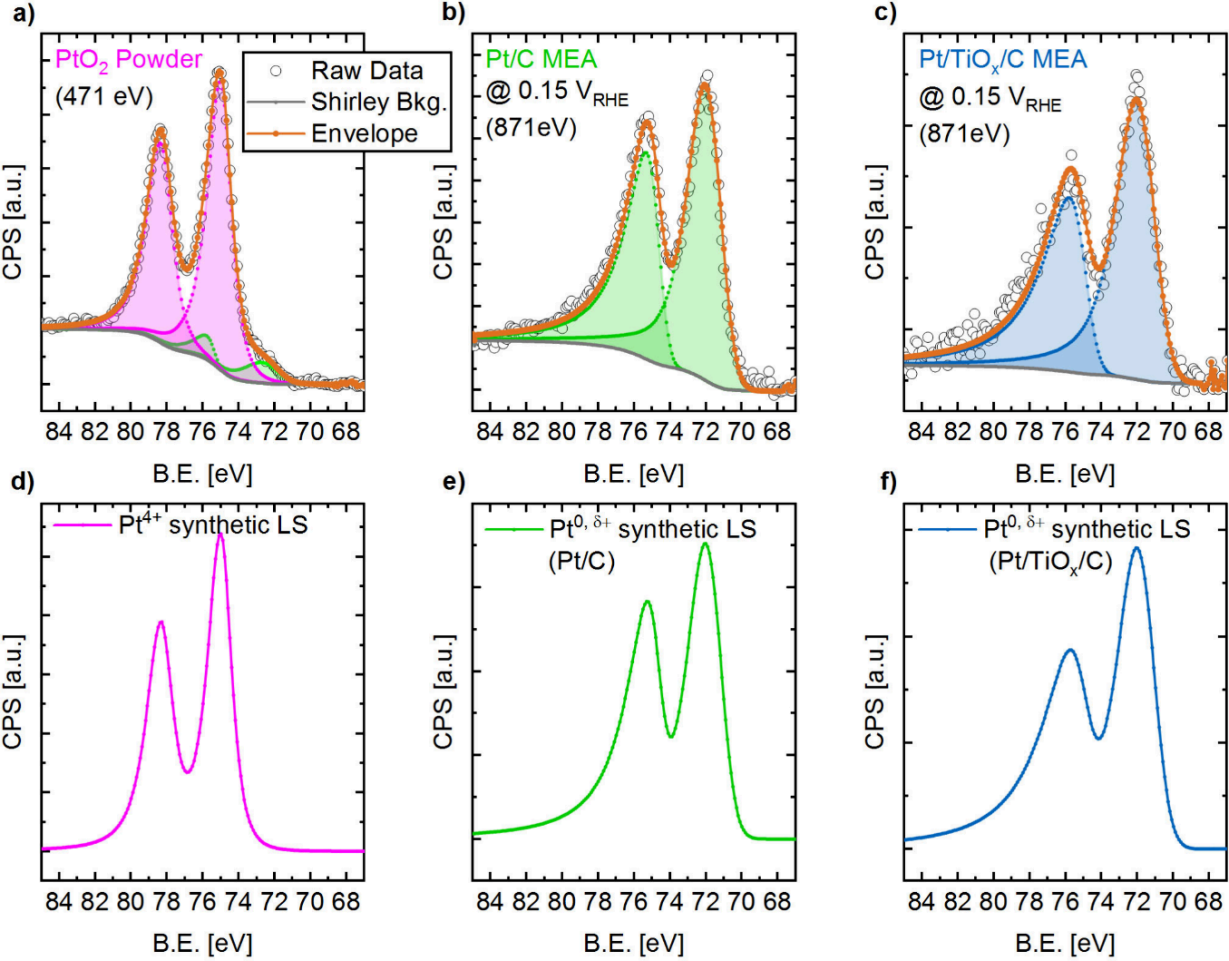
(a–c) C 1s energy-calibrated NAP-XPS Pt 4f spectra collected at ISISS/BESSY II on the reference samples and their peak models, which were used to generate synthetic line shapes. The orange dotted lines represent the envelope of the peak model, while the raw data are depicted as hollow black circles. A Shirley background is used throughout the study (gray line). The detailed peak model parameters are listed in Table 1. (a) PtO_2_ powder measured under UHV with an excitation energy of 471 eV (ex situ sample), serving as the Pt^4+^ and Pt^2+^ reference (pink line). The sample was not a pure Pt^4+^ reference, as indicated by the green line/area, representing a Pt^0, δ+^ species. Only this spectrum was not corrected for its binding energy, as almost no C 1s signal could be detected. (b,c) Pt/C and Pt/TiO_*x*_/C data, respectively, from MEAs in operando conditions at 0.15 V_RHE_ (H_UPD_ region, Pt^0^) under a dynamic vacuum of ≈0.02 mbar, using an excitation energy of 871 eV, 10 scans, 0.1 s_dwell_, and 0.1 eV/step in the Pt 4f region (86–66 eV binding energy); the corresponding peak models are green for Pt/C and blue for Pt/TiO_*x*_/C. (d–f) Synthetic line shapes derived from the peak models in (a–c) for Pt^4+^ (used also for Pt^2+^) (d), Pt^0, δ+^ from Pt/C as WE (e), and Pt^0, δ+^ from Pt/TiO_*x*_/C as WE (f).

The XPS data analysis was performed with CasaXPS (Version 2.3.25PR1.0). After binding energy (B.E.) calibration to the adventitious carbon signal (284.8 eV binding energy) in the C 1s region (for all operando spectra) and a Shirley-background subtraction of each XPS spectrum, a Lorentzian Asymmetric (LA) line shape was employed for the Pt 4f region to account for the asymmetry commonly observed for metals.^20,31–33^ A detailed description of the LA line shape, along with the fitting parameters used for the reference samples, is reported in the Section S3. For clarity, a brief overview of the key parameters of the LA­(α,β,ω) function is provided here.^32,34,35^ The parameters α and β control the slope of the Lorentzian tail for higher and lower binding energies, respectively, where higher values correspond to a steeper tail edge. Asymmetry is induced by introducing α ≠ β, which also affects the determined B.E. position according to the literature.^33,34^ The parameter ω is related to the contribution of the width of the Gaussian function, which is convoluted into the Lorentzian function. Using this line shape, the parameters (α, β, and ω) listed in Table 1 were used to describe a single individual peak of the Pt 4f doublets (either Pt 4f_7/2_ or Pt 4f_5/2_) corresponding to Pt^0^, Pt^2+^, and Pt^4+^ species as obtained from the reference sample spectra. The spin–orbit splitting between the Pt 4f_7/2_ and Pt 4f_5/2_ peaks is fixed at 3.34 eV if not mentioned otherwise, while it was made sure that no peak model was limited to any further constraints. For the upcoming discussion of the XPS data treatment and fitting, a differentiation between Pt^0^ and Pt^δ+^ (Pt with chemisorbed O) will not be made in this work since spectral deconvolution with fewer line shapes should provide a more robust result. Instead, Pt^0^ and Pt^δ+^ will be replaced with Pt^0, δ+^. Due to this simplification, relative B.E. positions may spread through a wider range between Pt^0^ and Pt^δ+^ (71.0–72.2 eV).

**Table 1:** Peak Model Parameters for Reference Samples Shown in Figure 3 Obtained Using the CasaXPS Software^a^

sample	component	line shape	B.E. position [eV]	fwhm [eV]	at %
PtO_2_ powder (ex situ, 471 eV excitation energy)	Pt^0, δ+^ 4f_7/2_	LA(1.2,50,100)	72.17	2.04	5.32
	Pt^0, δ+^ 4f_5/2_	LA(1.2,50,40)	72.17 + 3.34	0.98	3.99
	Pt^4+^ 4f_7/2_	LA(1.5,2.2,0)	75.02	1.42	51.83
	Pt^4+^ 4f_5/2_	LA(1.5,2.2,0)	75.02 + 3.34	1.54	38.87
Pt/C (operando at 0.15 V_RHE_, 871 eV excitation energy)	Pt^0, δ+^ 4f_7/2_	LA(1.2,50,100)	71.50	2	57.14
	Pt^0, δ+^ 4f_5/2_	LA(1.2,50,40)	71.50 + 3.34	1.6	42.86
Pt/TiO_*x*_/C (operando at 0.15 V_RHE_, 871 eV excitation energy)	Pt^0, δ+^ 4f_7/2_	LA(1.2,50,100)	71.42	2.29	57.14
	Pt^0, δ+^ 4f_5/2_	LA(1.2,50,40)	71.42 + 3.75	2.06	42.86

a LA line shapes were used according to the equations described in the CasaXPS manual.^34^ The line shapes of the Pt^0, δ+^ species were derived from the operando measurement of Pt/C and Pt/TiO_*x*_/C at 0.15 V_RHE_, whereas the line shape of Pt^4+^ was obtained from ex situ data of a PtO_2_ powder sample. The Pt^4+^ line shape was used for the Pt^2+^ species, as described later. The PtO_2_ powder did not receive any pretreatment prior to measurement and was used in its pristine state. Each peak of the Pt 4f doublet, namely, Pt 4f_7/2_ and Pt 4f_5/2_, is represented by an individual LA line shape.

As seen in Figure 3, the resulting envelopes (orange dotted line) based on the LA line shapes align well with the raw data. As generally anticipated from the expected electrochemical behavior due to H_UPD_, no Pt oxidation at 0.15 V_RHE_ was observed for both operando MEAs, since only a sole Pt 4f doublet with Pt 4f_7/2_ ≈ 71.5 eV, representative for a Pt^0, δ+^ species, is visible. Their B.E. position is higher than expected for a pure Pt^0^ phase, e.g., 70.9–71.1 eV reported by Mom et al.;^20^ this could be explained by the incorporation of ionomer in the catalyst layer, where sulfonate groups adsorb to Pt, leading to a higher contribution by Pt^δ+^. A notable difference between data from the Pt/C and Pt/TiO_*x*_/C samples is evident. In the latter case, a higher spin–orbit splitting of 3.75 eV (compared to 3.34 eV) was necessary to accurately represent the raw data. This alteration may be attributed to the overlap with a Ti 3s plasmon (≈74–75 eV) from the TiO_*x*_ support, as demonstrated by a model system by Petzoldt et al.^36^ This contribution is determined to be relatively minor (≈1–2% of the Pt 4f area, see the Section S3 and Figure S4). However, Petzoldt et al. also showed that the SMSI effect does not affect the spin–orbit splitting.^36^ This discrepancy in the fitting of spin–orbit splitting between the two samples could come from the difference in signal-to-noise ratios, much lower in the case of Pt/TiO_*x*_/C. Nevertheless, we used the line shape of the Pt/TiO_*x*_/C sample at 0.15 V_RHE_ to represent the Pt^0, δ+^ species for the corresponding XPS operando data set.

The PtO_2_ powder received no prior pretreatment and was measured in its pristine state, thus showing no pure Pt^4+^ (Pt 4f_7/2_ ≈ 75.0 eV) phase but also another Pt 4f doublet, which is likely ascribed to chemisorbed oxygen on Pt^δ+^ (Pt 4f_7/2_ ≈ 72.2 eV).^15^ Given that a sole Pt^2+^ species does not exist in a stable and pure form, direct experimental determination of its line shape is not feasible, as experiments in regards to the thermal stability of PtO_2_ show the reduction into either a mixed phase of different oxidation states or directly into metallic Pt.^37–39^ Consequently, we adopt the Pt^4+^ line shape, which is closer in oxidation state to Pt^2+^, as a reasonable approximation.^40^ Although Pt^4+^ is more oxidized than Pt^2+^, their electronic environments share similarities, particularly in terms of core-level binding energies and chemical shifts associated with higher oxidation states. To account for the possible uncertainty in the true Pt^2+^ line shape, as well as for any binding energy shifts due to charging effects, we slightly loosened the Pt^4+^ and Pt^2+^ positions and full width at half-maximum (fwhm) constraints compared to the Pt^0, δ+^ species. The variations in peak position and fwhm for all the operando data sets can be seen in Section S4. While an approximation, this approach is a practical solution within the limits of XPS and ensures a reliable representation of all of the oxidized Pt species in the resulting fit. Further refinements based on advanced simulations or complementary techniques may improve this approximation. However, for the scope of our study, using the Pt^4+^ line shape also as for Pt^2+^ provides the most accurate and reproducible framework for Pt^2+^ analysis.

Using these determined LA line shapes as references, synthetic line shapes based on the envelope of the Pt 4f_7/2_ and Pt 4f_5/2_ peaks of a single Pt 4f doublet are created for each Pt species, as shown in Figure 3d–f, forming the basis for further data analysis of the operando NAP-XPS spectra. These synthetic line shapes are used throughout this study to simplify the spectral deconvolution (see Tables S2–S4). This approach provides a more robust data fitting, particularly regarding the fluctuating fwhm values for both Pt 4f_7/2_ and Pt 4f_5/2_ peaks, which are now represented by a single fwhm scaling factor reported in Table S4. The peak positions of synthetic line shapes listed in Table S2 and Table S4 differ from the determined B.E. positions through LA functions because they are defined as the B.E. values at the peak maximum of Pt 4f_7/2_, due to the simplification of fitting the peaks not with a doublet but with one single component. Based on the results of the synthetic line shapes, the area for each Pt oxidation state at the investigated potentials is then determined to derive the at % of each specific state of the Pt particle. To be able to compare XPS and electrochemical results, the following equation is required to ensure a consistent unit of e^–^ Pt_sa_^–1^ associated with Pt oxidation:

3 $$\frac{{n_{e^{-}}}^{XPS,{Pt}^{2+}}+{n_{e^{-}}}^{XPS,{Pt}^{4+}}}{{Pt}_{sa}}=\frac{e^{-}}{{Pt}_{sa}}$$

Here, *n*_e^–^_^XPS,Pt^2+/4+^^ represents the number of electrons detected via XPS, determined and based on the at % of Pt^2+/4+^ species, considering either two or four electrons transfer from Pt^0, δ+^ for each oxidation state. This calculation was based on the total number of Pt atoms in a 3 nm bulk particle. To obtain a good approximation of the XPS information depth (defined as the thickness from which 95% of the photoelectron signal is detected), we calculated the signal attenuation with the Lambert–Beer law modified for XPS (see Section S4), using the expected thicknesses and the effective attenuation lengths (EALs, at the kinetic energy of 800 eV) for Pt and each overlayer (BLG, ionomer, and SMSI-induced TiO_*x*_ layer on Pt/TiO_*x*_/C), obtained using the NIST Electron Effective-Attenuation-Length Database (software version 1.3) and reported in Section S4.^41^ The EAL calculations are based on the Tanuma, Powell, and Penn (TPP-2M) model.^42^ The resulting information depths were about 1.7 and 1.55 nm for Pt/C and Pt/TiO_*x*_/C, respectively, roughly half the diameter of the Pt particles and thus with an identical surface-to-bulk ratio compared to the entire particle. Based on this, we can be confident that the atom % of the Pt species obtained by the XPS fitting are representative of the entire particle, enabling direct comparison to the electrochemical data by normalizing the detected electrons to the number of surface atoms (e^–^ Pt_sa_^–1^), and ensuring consistency in interpreting the Pt oxidation behavior.

### 2.6 Transmission Electron Microscopy (TEM)

To gain further insights into the deposition of the Pt particles in the case of the Pt/TiO_*x*_/C catalyst powder, transmission electron microscopy (TEM) was performed using a JEM-1400 Plus microscope (JEOL, Germany) with an accelerating voltage of 120 kV and a magnification of 100k. For this purpose, the catalyst powder was dispersed and sonicated for 5 min in acetone (≥99.9% suitable for HPLC, Sigma-Aldrich, Germany) and drop-casted onto a TEM grid (Formvar/Carbon Square Mesh, Cu 400 Mesh, Electron Microscopy Sciences, USA).

## 3 Results and Discussion

### 3.1 Electrochemical Validation of the Operando Cell

To validate the operando cell, CVs were measured to assess the electrochemical behavior of the catalysts and compare the CVs with the literature data. Figure 4 shows one exemplary CV measured for the MEAs containing Pt/C (green line) and Pt/TiO_*x*_/C (blue line) as WEs. For the Pt/C catalyst, all distinctive and characteristic features in their respective regions, namely the H_UPD_ region (<0.45 V_RHE_) and the Pt-oxide region (>0.75 V_RHE_), can be identified. Regarding the Pt/TiO_*x*_/C catalyst, the H_UPD_ region is reduced due to the SMSI-induced TiO_*x*_ overlayer on top of the Pt particles. This layer is described in the literature^5,8^ as an overlayer that is permeable for hydrogen or protons, as reported by Hsieh et al.^4^ and further supported by RDE experiments by Stühmeier et al.,^3,9^ who reported a decent HOR/HER activity (≈0.33 times the activity of pure Pt/C) of the Pt/TiO_*x*_/C catalyst. Notably, the Pt-oxide region is strongly suppressed by this overlayer, in agreement with reports stating the impermeability of any oxygenated species to this overlayer, while maintaining HOR/HER activity in RDE experiments, even at potentials as high as 1.5 V_RHE_, at which a deactivation due to Pt-oxide formation would have been normally expected.^3^ This observation demonstrates that Pt with an SMSI-induced layer such as TiO_*x*_ does not oxidize under those conditions. It is important to note that although minimal, some current from the Pt-oxide formation for Pt/TiO_*x*_/C can be observed in Figure 4, indicating the presence of Pt particles located also on the carbon instead of solely on TiO_*x*_, thus missing the SMSI-induced layer. Indeed, TEM micrographs of the catalyst powder, as shown in Figure 5, confirm the existence of such particles supported on carbon rather than on TiO_*x*_.

**Figure 4:**
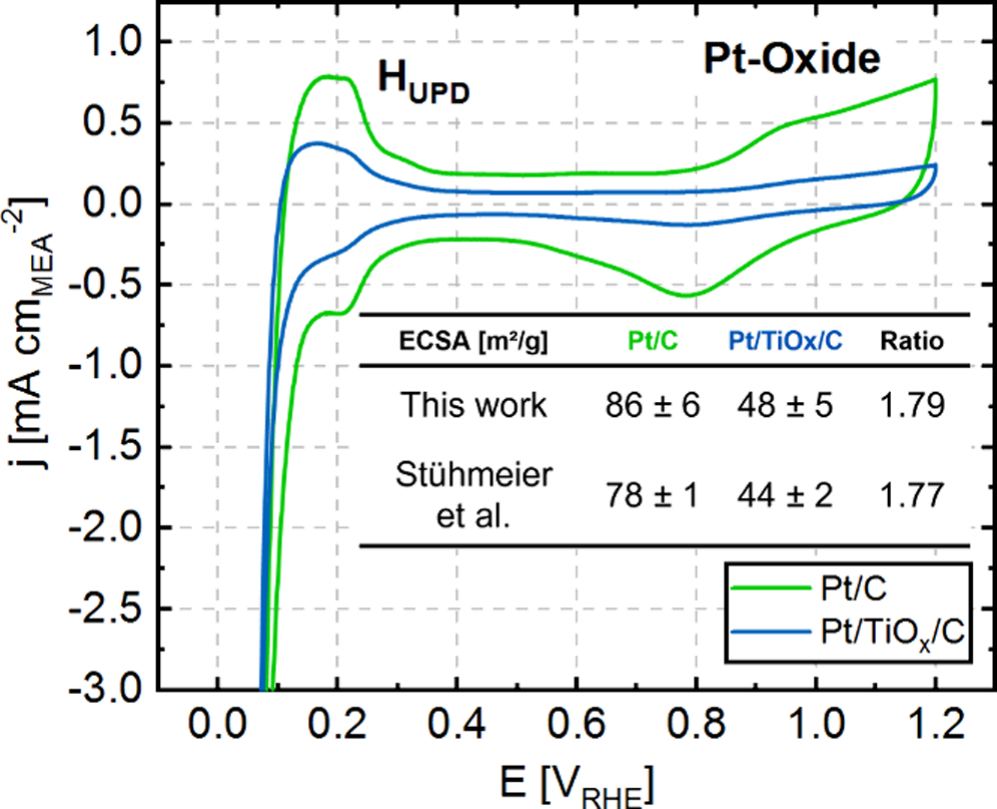
Cyclic voltammograms (CVs) at 10 mV s^–1^ between 0.045 and 1.2 V_RHE_, collected using the MEAs with Pt/C (green) or Pt/TiO_*x*_/C (blue) as WEs (with Pt loadings of ≈45 ± 5 μg_Pt_ cm_MEA_^–2^) in the operando cell at ISISS/BESSY II (see Figure 1) at RT and under a dynamic vacuum of ≈0.02 mbar in the WE compartment. The inset table reports the ECSA of each catalyst determined and averaged across multiple scan rates (150, 100, 50, and 10 mV s^–1^). Since the catalyst layers are the same as reported in literature, the ECSAs were estimated by integrating the H_UPD_ area (0.090–0.45 V_RHE_) and compared well with those measured in a 5 cm^2^ PEMFC single-cell by Stühmeier et al.^9^

**Figure 5:**
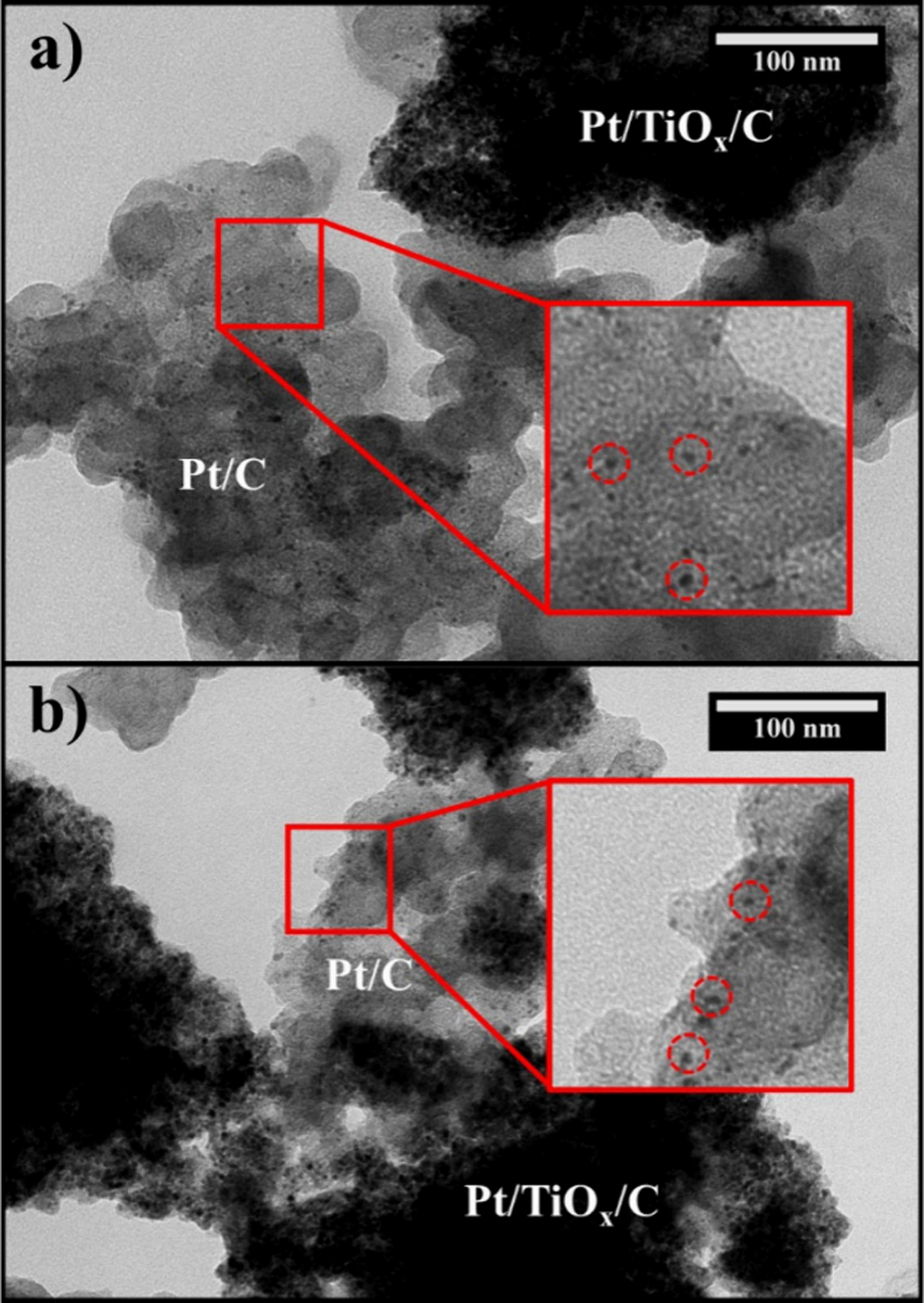
Two exemplary TEM micrographs of the Pt/TiO_*x*_/C catalyst powder at a magnification of 100k and an accelerating voltage of 120 kV. Dark clusters indicate agglomerates of Pt/TiO_*x*_/C, whereas lighter areas depict carbon support with Pt particles visibly deposited. (a) Catalyst region predominantly populated by carbon support and (b) catalyst region predominantly populated by TiO_*x*_/C support, showing examples of the entire catalyst structure. Zoomed-in parts of the picture are represented in the red inset, where Pt nanoparticles are highlighted by red dashed circles.

Darker clusters in the TEM micrographs represent the Pt/TiO_*x*_/C catalyst, with an accumulation of Pt particles, TiO_*x*_, and the carbon support. In contrast, lighter areas predominantly show carbon support, on which Pt nanoparticles are also clearly deposited. Therefore, the results presented in this study for the Pt/TiO_*x*_/C catalyst are partially affected by the presence of unprotected Pt/C particles. However, this does not impact the conclusions regarding the effect of the SMSI-induced TiO_*x*_ layer on this catalyst, as will be demonstrated by the results of the operando NAP-XPS data. To further validate the operando cell setup, the electrochemical surface area (ECSA) of each catalyst was quantified, yielding values similar to those obtained by Stühmeier et al., as shown in the inset table of Figure 4.^9^ Although the ECSA values obtained from the operando cell are slightly higher (≈10%), the relative ECSA ratio between both catalysts is virtually equal to those measured in a 5 cm^2^ single-cell PEMFC, confirming the reliable electrochemical performance of the optimized operando cell design, even though the RH condition of the operando cell cannot be controlled perfectly due to the vacuum applied in the measurement chamber. The Pt-oxide features in the CVs were selected as a primary indicator to estimate the RH level at the catalyst layer in the operando cell due to the RH-dependence of the sensitivity of the overpotential for oxide formation.^26^ Figure S3 compares the CVs obtained from the operando cell with CVs from a 5 cm^2^ single-cell PEMFC using an MEA with the same WEs at different RHs. This indicates that the RH of the WE in the operando cell is lower than 40% RH but definitely higher than 0% RH, demonstrating that the complete dry-out of the WE does not occur.

### 3.2 Operando NAP-XPS Data

The obtained operando NAP-XPS data using MEAs with Pt/C and Pt/TiO_*x*_/C as WEs are shown in Figure 6. Qualitatively, Pt/C exhibits no oxidation at potentials between 0.15 and 0.45 V_RHE_, which is consistent with what one would expect for potentials in/near the H_UPD_ region (see Figure 4). Pt oxidation begins at potentials ≥ 1.0 V_RHE_, consistent with the appearance of a shoulder in the Pt 4f_5/2_ region at ≈78–80 eV for potentials of 1.0 and 1.2 V_RHE_, which evolves into a clearly visible peak at ≈78 eV for 1.4 V_RHE_ (see Figure 6a); the latter can be ascribed to the Pt^4+^ 4f_5/2_ feature. In contrast, Pt/TiO_*x*_/C barely oxidizes upon increase of the potential even to 1.4 V_RHE_, largely remaining in the Pt^0, δ+^ state (see Figure 6b), with only rather minor changes in the XPS spectrum at 1.4 V_RHE_ compared to that at 0.15 V_RHE_ in the 78–80 eV range characteristic for Pt^4+^. This observation aligns with the electrochemical data and confirms that the Pt particles in the Pt/TiO_*x*_/C catalyst exhibit minimal oxidation, likely due to the Pt nanoparticles partially deposited on carbon instead of on TiO_*x*_. Concerning the detection of any possible electronic interaction between Pt and the encapsulating TiO_*x*_, knowing that the catalyst consists of 21.6 wt % Ti or 46 wt % TiO_2_, using the Pt particle diameter (3 nm), the mass percentage of Pt in the catalyst (15 wt % Pt), and assuming that the Pt encapsulation consists of one monolayer of TiO_2_, we can estimate that only at most ≈3% of the TiO_2_ support could contribute to this effect. This amount would be negligible in Ti 2p XPS data, making detection impossible. The case of XPS detection using O 1s is complicated even further due to the presence of oxygen containing ionomer in the whole catalyst layer.

**Figure 6:**
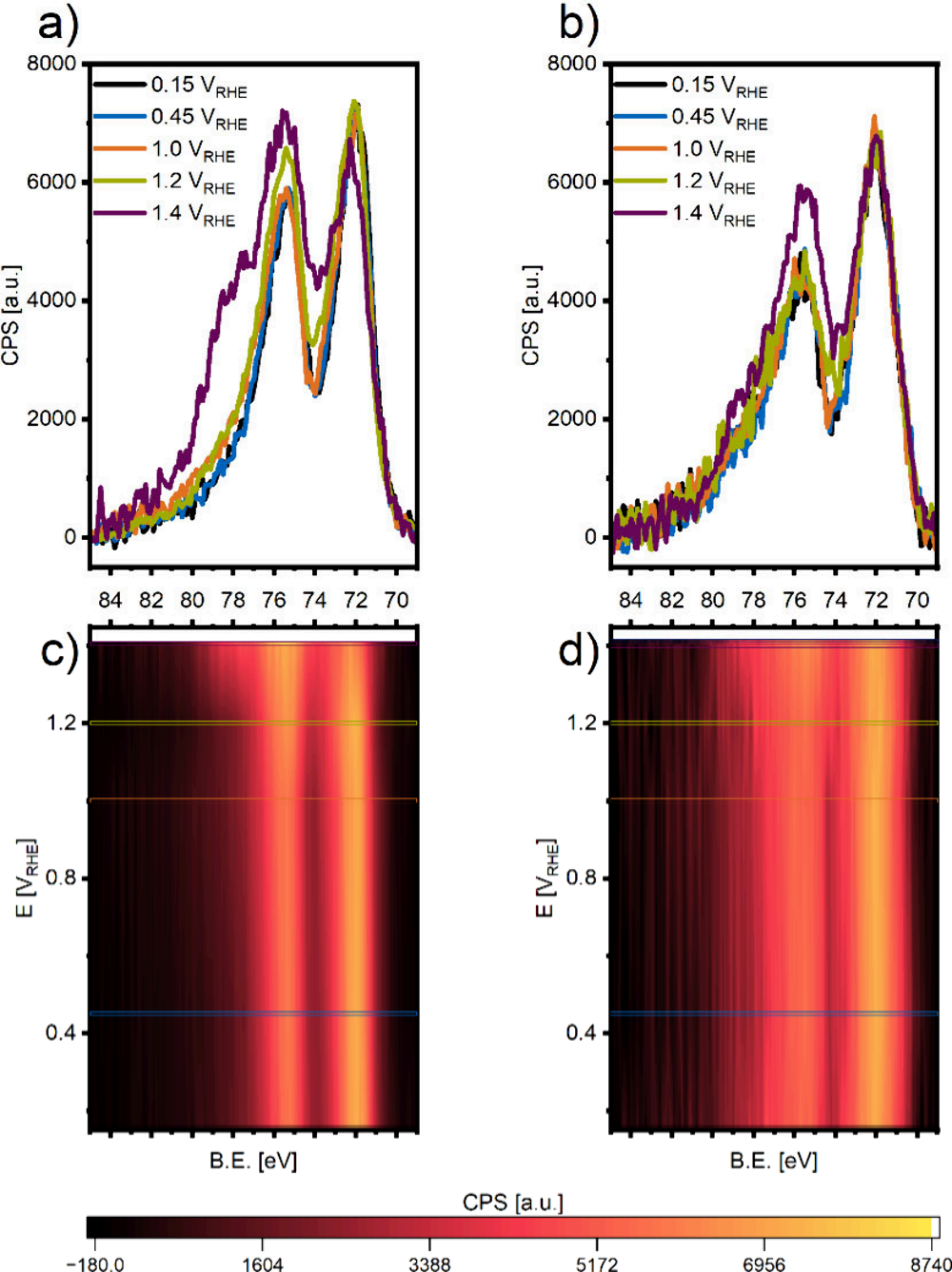
NAP-XPS Pt 4f spectra of MEAs with Pt/C (a) and Pt/TiO_*x*_/C (b) as WEs in the operando cell at various potentials, after Shirley background subtraction, C 1s energy calibration, and area normalization to the Pt^0, δ+^ species. The spectra were collected in the Pt 4f region (86–66 eV binding energy) under a dynamic vacuum of ≈0.02 mbar, using an excitation energy of 871 eV, 10 scans, 0.1 s_dwell_, 0.1 eV/step. (c,d) Contour plots of the operando spectra as a function of potential. The horizontal lines indicate the potentials at which the spectra shown in (a) and (b) are measured. CPS is used for the color coding range from low intensity (black) to high intensity (yellow), visualizing changes in the operando spectra with potential.

To further quantify this phenomenon, each operando data set is fitted using the synthetic line shapes derived from the reference samples (see the experimental part in Section 2.5) to determine the atomic ratio of different Pt oxidation states for each catalyst. The resulting fit of the XPS data is exemplary and shown for 1.0 and 1.4 V_RHE_ in Figure 7, visualizing features of Pt^0, δ+^, Pt^2+^, and Pt^4+^. At 1.0 V_RHE_, a minor presence of Pt^4+^ (pink dotted lines) instead of Pt^2+^ is evident for both catalysts (see Figure 7a,c), which requires further explanation. According to extensive studies with various setups and techniques (mostly in liquid electrolytes) reviewed by Conway et al., it is hypothesized that Pt hydroxide species are initially adsorbed as a submonolayer, which may lead, via a possible place-exchange process, to the formation of a stoichiometric average of “PtO” (or Pt^2+^) that further oxidizes into a more bulk-type oxide “PtO_2_” (or Pt^4+^).^28^ However, these studies do not include any additional effect on the Pt oxidation by a perfluorosulfonic acid (PFSA) ionomer, used both here and in the work by Mom et al., enabling the comparisons between the two studies.^20^ Using a similar operando cell setup, Mom et al. sputtered Pt nanoparticles onto an alkaline and an acidic membrane (FAD55 and Nafion 117, respectively) and analyzed the Pt-oxide formation of those particles with increasing potential.^20^ Major differences in cell setups between this work and Mom et al.^20^, possibly impacting the comparison, are the way to supply water (humidified gas vs liquid aqueous electrolyte), the overall electrical resistance acquired via EIS (≈5 Ω vs 40–50 Ω, likely due to contact resistance at the sputtered Pt nanoparticle interface and the position of the reference electrode in the operando cell from Mom et al.), and the catalyst system (employed catalyst layer with ≈6–8 μm thickness vs an at most 16 nm sputtered Pt film as model system). The humidification using liquid water instead of humidified gas certainly improves the wetting of the sputtered Pt in the case of Mom et al. However, this applies well only to the Pt nanoparticles right in the vicinity of the membrane and then relies on the effectiveness of the BLG deposition to retain a thin liquid film across the whole thickness of the sputtered electrode. In contrast, the catalyst layer used in this work has a dispersed ionomer network and, therefore, is able to provide adequate wetting and efficient proton conductivity throughout the whole catalyst layer thickness, with an average RH between clearly above 0% and less than 40%.^20^ These are also the reasons for the much improved CV data of the operando cell in this work, which nicely reproduce those in a real PEMFC. In the work by Mom et al., with an acidic Nafion 117 membrane, both Pt^2+^ and Pt^4+^ were found at 1.15 V_RHE_, corroborating the general observations reported in the literature.^20,28,43,44^ In this work, the spectrum at 1.0 V_RHE_ does not indicate the presence of any Pt^2+^, which only emerges at the higher potentials of 1.2 and 1.4 V_RHE_ (see Table S2). Based on the spectral deconvolution at 1.0 V_RHE_, including a Pt^2+^ species was considered unnecessary, as the XPS data are accurately described with only Pt^0, δ+^ and Pt^4+^ species. Introducing a Pt^2+^ species (see Table S4, Pt 4f_7/2_ ≈ 74.3 ± 0.2 eV) would have resulted in an overrepresentation of the valley around ≈73.5–74.5 eV in the envelope in Figure 7a, whereas the shoulder formation at ≈78–80 eV is clearly attributed to the Pt 4f_5/2_ feature of the Pt^4+^ species. Still, the potential formation of minute amounts of Pt^2+^ in the spectra cannot be ruled out. The Pt/TiO_*x*_/C catalyst, even though barely any oxidation is observed, displays a similar behavior at 1.0 V_RHE_ (see Figure 7d and Table S3), requiring Pt^4+^ but no Pt^2+^ species. The oxidation behavior likely originates from the Pt particles deposited on carbon instead of TiO_*x*_ (as shown in the TEM images of Figure 5). Detailed information about the synthetic line shapes for each potential and catalyst is shown in Tables S2–S4.

**Figure 7:**
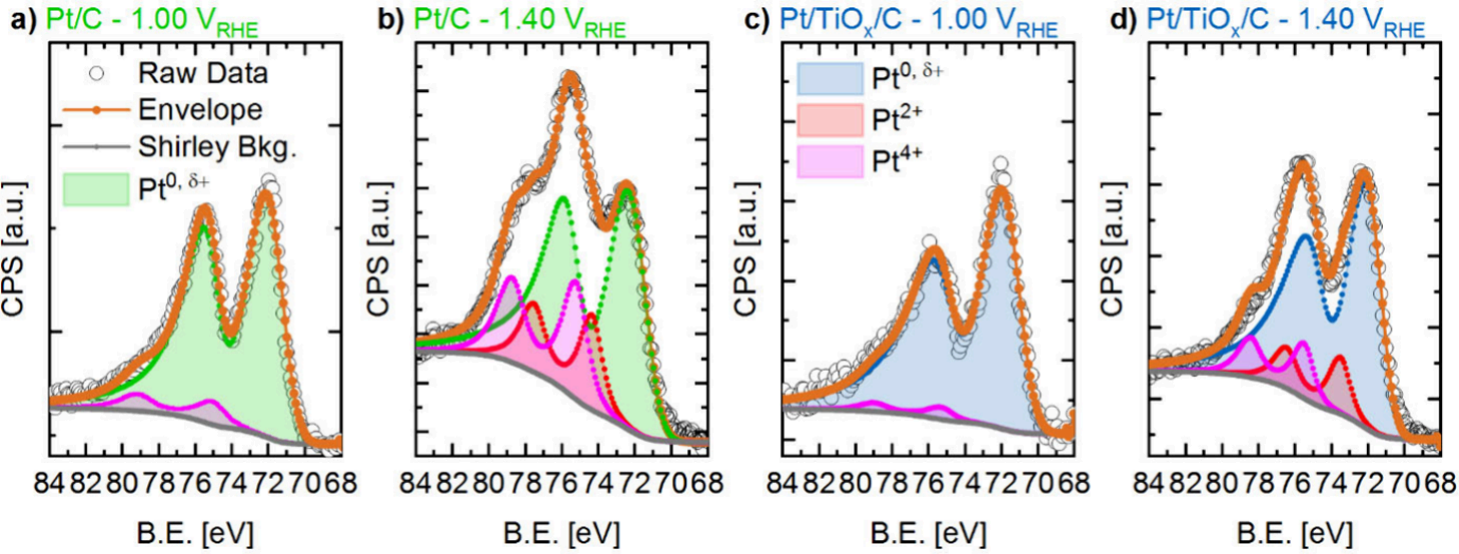
Exemplary Pt 4f NAP-XPS spectra after C 1s energy calibration of MEAs with Pt/C (a,b) and Pt/TiO_*x*_/C (c,d) as WEs, measured in the operando cell at 1.0 and 1.4 V_RHE_, respectively. The spectra were collected in the Pt 4f region (86–66 eV binding energy) under a dynamic vacuum of ≈0.02 mbar, using an excitation energy of 871 eV, 10 scans, 0.1 s_dwell_, 0.1 eV/step. The acquired raw data are shown in hollow dots, while the Shirley background is shown as a gray solid line. The resulting envelopes of the components (orange solid lines) are obtained using Pt^0, δ+^ (green dotted), Pt^2+^ (red dotted), and Pt^4+^ (pink dotted) species characterized by the synthetic line shapes derived from reference samples (see the experimental part).

Using the obtained at % for the different Pt oxidation states from the fitted spectra, the number of electrons associated with Pt oxides per Pt surface atom (e^–^ Pt_sa_^–1^) can be calculated from XPS according to eq 3; on the other hand, the e^–^ Pt_sa_^–1^ value can also be calculated from electrochemical data using eqs 1 and 2. The results of these calculations are presented in Figure 8.

**Figure 8:**
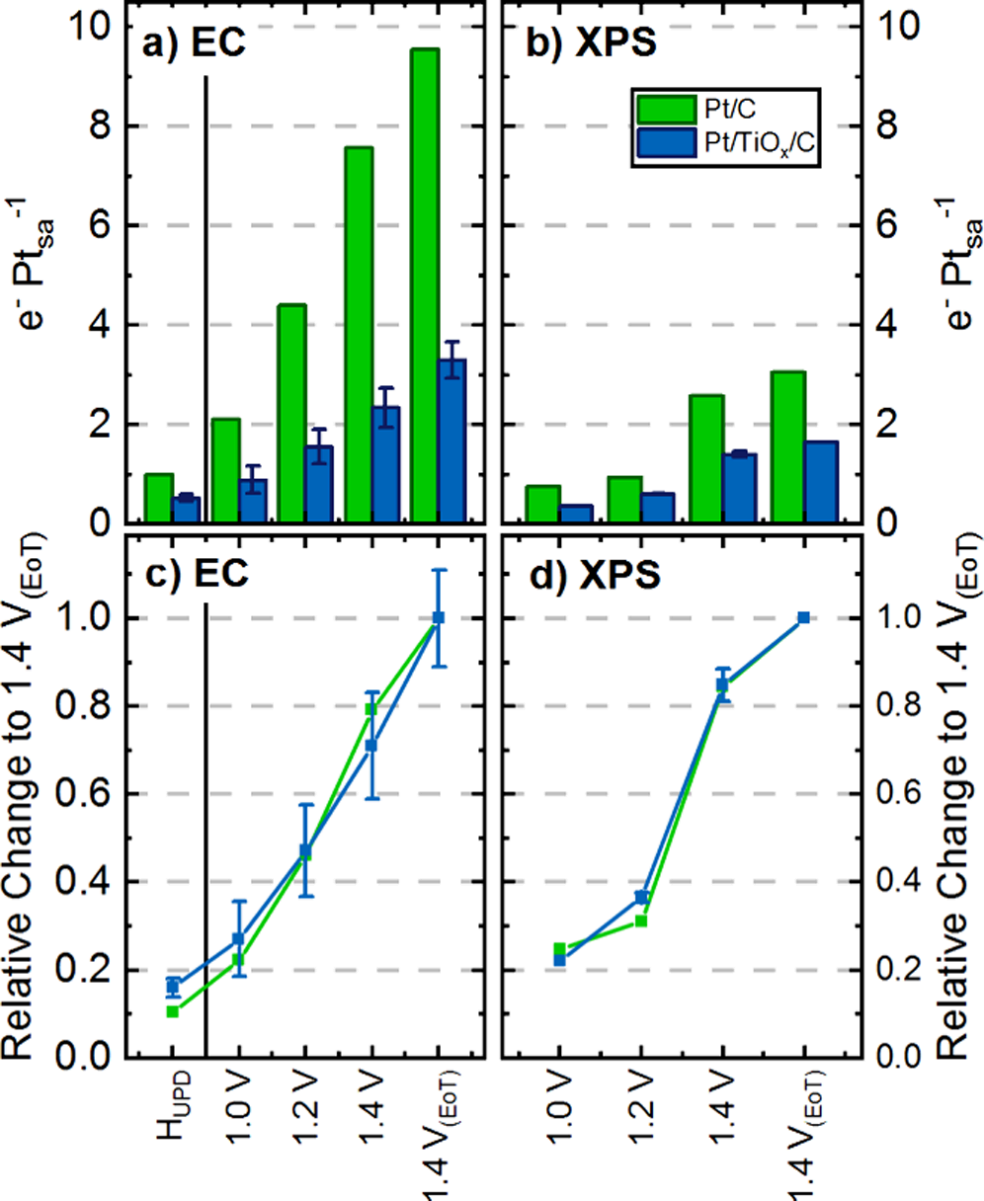
Calculated potential-dependent electrons for Pt oxidation, normalized to the Pt surface atoms (Pt_sa_), using (a) the electrochemical data and (b) the operando NAP-XPS spectra of Pt/C (green) and Pt/TiO_*x*_/C (blue). In (a), the electrons calculated from 1.0 V_RHE_ onward are added up. Their relative change normalized to the value at 1.40 V_RHE_ (EoT) are shown respectively in (c) for the electrochemical data and (d) for the operando NAP-XPS spectra. In addition to Pt oxidation, the electrons associated with the H_UPD_ features from Figure 4 were added in (a) and (c) as a reference to one monolayer of adsorbed H (or one electron per Pt_sa_). The error bars are derived using two measurements of MEAs with Pt/TiO_*x*_/C as the WE, while for Pt/C only one measurement was performed. The error bars in (b) and (d) also originate from two measurements, which include a variable surface dry-out at different measured spots due to the varying number of cracks in the deposited BLG film within the beam size of 100 × 300 μm.

In addition to the Pt oxidation, the electrons associated with the H_UPD_ region are also calculated from the charges observed in the CV data in Figure 4 and are included in the electrochemical analysis, as shown in Figure 8a,c, in order to provide a value representing one monolayer of an adsorbed species on Pt. As known from the regular electrochemical behavior of Pt, Pt/C delivers 1 e^–^ Pt_sa_^–1^ in the H_UPD_ area, corresponding to one hydrogen atom adsorbed per Pt surface atom.^5,45^ In contrast, Pt/TiO_*x*_/C exhibits ≈0.5 e^–^ Pt_sa_^–1^ due to the aforementioned SMSI-induced TiO_*x*_ overlayer encapsulating the Pt particle (left most green and blue bar in Figure 8a). Regarding Pt oxidation at potentials from 1.0 to 1.4 V_RHE_, an increase in e^–^ Pt_sa_^–1^ is observed upon a potential increase (see Figure 8a), as also qualitatively observed in the NAP-XPS spectra shown in Figure 6 and Figure 7 for both catalysts. For Pt/C, ≈2 e^–^ Pt_sa_^–1^ are associated with Pt oxides at 1.0 V_RHE_, whereas ≈9 e^–^ Pt_sa_^–1^ are calculated at 1.4 V_RHE_ (blue bars in Figure 8a). Values exceeding 4 e^–^ Pt_sa_^–1^ correspond to an overstoichiometric average of “PtO_2_”, suggesting the formation of more than one oxidic monolayer. The results differ from literature reports such as those by Angerstein-Kozlowska et al., where 1.0 e^–^ Pt_sa_^–1^ and 1.9 e^–^ Pt_sa_^–1^ were found for the aforementioned potentials, respectively.^43^ The differences arise from the experimental setups, conditions, measurement procedures and evaluation, as Angerstein-Kozlowska et al. used bulk polycrystalline Pt in a liquid-phase setup and derived the number of electrons based on the charge observed solely from CVs, whereas this study employed ionomer-bonded electrodes with carbon-supported Pt nanoparticles and sequential potential holds to enable operando NAP-XPS measurements.^43^ If solely the LSV charges are taken into consideration (see Figure 2, yellow areas), the values are more consistent with literature (≈0.7 and 1.5 e^–^ Pt_sa_^–1^ for 1.0 and 1.4 V_RHE_, respectively),^28,43^ while minor deviations are attributed to differences in RH and consequent overpotentials in the Pt-oxide formation between the setups. When comparing the two investigated catalysts to each other, it becomes apparent from Figure 8a that the e^–^ Pt_sa_^–1^ values obtained for Pt/TiO_*x*_/C are ≈40% than those for the oxide formation process in Pt/C across all potentials. In addition to the evaluation of absolute e^–^ Pt_sa_^–1^ values, relative changes with respect to the EoT values are shown in Figure 8c. The Pt-oxidation behavior as a function of potential appears to be identical between the two catalysts. Since Pt/C is used as a reference, this confirms that in Pt/TiO_*x*_/C a significant fraction of the Pt particles of this catalyst must be supported on carbon instead of TiO_*x*_, as already evidenced previously in the TEM images of the catalyst powder in Figure 5. Using the extent of Pt oxidation as a primary indicator, while assuming no oxidative contribution of the TiO_*x*_-encapsulated Pt particles, an estimate of the Pt/C fraction can be made based on the electrochemical data. As stated before based on the electrochemical data of Pt/TiO_*x*_/C, an average of ≈40% of Pt_sa_ experiences oxide formation compared to Pt/C, which corresponds well to the observed ORR mass activity in a PEMFC single cell measurement by Stühmeier et al. (≈33% vs Pt/C).^9^ This suggests that around 33–40% of Pt particles are supported on carbon in the Pt/TiO_*x*_/C catalyst. Examining the data extracted from the NAP-XPS spectra in Figure 8b, the general trend of Pt-oxide formation with increasing potential is also confirmed, since Pt/TiO_*x*_/C retains ≈55% of the Pt oxide vs Pt/C across all potentials.

On the other hand, comparing Figures 8a and 8b, a huge discrepancy is evident between the absolute values of electrochemical and XPS results. The most plausible explanation for this discrepancy is the likely presence of numerous cracks in the BLG film used in the operando cell, allowing water to evaporate at the surface below the cracks.^11^ This, in turn, results in a significant fraction of the Pt particles within the XPS detection depth that have no access to the reactant (water) that is required for Pt particle surface oxidation. According to Mom et al., on their sputtered Pt electrode, the BLG-film main purpose is to provide electrical contact to isolated nanoparticles (irrelevant for our Pt/C WE, since the Pt nanoparticles are electrically well-contacted via the carbon support) and to act as an evaporation barrier, as some degree of humidity is necessary to oxidize Pt.^11,20^ The BLG does not influence dramatically the overall electrochemistry, as demonstrated in Figure S1, where CVs of an MEA using Pt/C as WE with and without BLG show similar redox features, suggesting that the cracks in the BLG affect only the particles on the electrode surface, which is negligible in amount compared to the bulk electrode probed by electrochemistry. Furthermore, the electrochemical data demonstrate that the applied dynamic vacuum of ≈0.02 mbar in the measurement chamber is insufficient to completely dry out the bulk of the electrode, since this would otherwise result in zero current in the CV (see Figure S3). Thus, it is reasonable to assume that water can reach the bulk of the electrode. The transport of water through an ionomeric membrane (as well as through the ionomer film within the electrodes) is governed by water diffusion if a water activity gradient is present.^46,47^ In this work, specifically, the vacuum creates an additional pressure differential that draws water toward the measurement chamber. The highest water transport resistance, according to Duan et al., is at the membrane/vapor interface, where water goes into the vapor phase.^46,47^ Consequently, applying a pressure differential, the region of the membrane/ionomer with the lowest water content (thus, the lowest effective RH) is expected at the ionomer/vacuum interface, i.e., essentially within the XPS detection depth. The BLG effect is further illustrated in Figure S2, which compares the Pt 4f region measured for an MEA with Pt/C as WE with and without BLG, demonstrating clearly that the BLG is necessary to observe Pt oxidation when a potential of 1.4 V_RHE_ is applied. It becomes evident that the BLG is certainly keeping the electrode surface humidified, enabling Pt oxidation, but the extent of this retained surface humidification is related to the extent of cracks in the BLG and to water surface diffusion, which likely leads to the discrepancy observed between Figures 8a (EC) and 8b (XPS). The structural integrity of the BLG can be estimated by taking the ratio between e^–^ Pt_sa_^–1^ from XPS (Figure 8b) and e^–^ Pt_sa_^–1^ from EC (Figure 8a, considered a good estimate of the real total e^–^ Pt_sa_^–1^) within one sample at the same potential. This ratio represents the fraction of electrode surface protected by an intact BLG in the experiment, resulting in 39 ± 11% across all measured samples and potentials. Furthermore, the error bars depicted in Figure 8b,d also stem from the statistically variable number of cracks in the BLG within the beam size of 300 × 100 μm (width × height). Even though this discrepancy exists, a closer look into the relative values in Figure 8d still provides insights into the effect of the SMSI-induced TiO_*x*_ layer: similarly to the relative trend of electrochemical data upon potential increase (see Figure 8c), the analysis of the relative variation in NAP-XPS operando data on Pt/TiO_*x*_/C as a function of potential closely follows the relative trend of the Pt/C (Figure 8d).

In general, the data in Figure 8 supports the conclusion that, with the electrochemically optimized setup used in this study, the effect of an SMSI-induced TiO_*x*_ layer can also be observed using operando NAP-XPS, i.e., that Pt oxidation is demonstrated to be significantly suppressed even at the high potential of 1.4 V_RHE_, confirming the observed trend in the electrochemical data reported in Figure 4 and explaining the high and stable HOR/HER performance up to potentials of 1.5 V_RHE_ in RDE experiments.^3,9^ Since the residual oxidation of the Pt/TiO_*x*_/C catalyst is likely due to Pt particles deposited on carbon, a pure phase of Pt/TiO_*x*_/C with an oxidation-preventing SMSI-induced TiO_*x*_ layer is expected to barely oxidize under the investigated potential range, leading to a vastly different potential dependence of the Pt oxidation compared to Pt/C and thus superior hydrogen selectivity, which enhances durability in SUSD events.

## 4 Conclusions

A previously designed operando NAP-XPS cell from Velasco-Veléz et al.,^11,18,20,22^ using BLG as an X-ray transparent window with very little beam attenuation, is implemented and optimized for the use of PEMFC-like MEAs without liquid electrolyte for the first time at the ISISS workstation of BESSY II, achieving electrochemical measurements very similar to those collected in a standard single-cell PEMFC. This was verified by collecting CVs of two catalysts, namely Pt/C and Pt/TiO_*x*_/C, at the WE under Ar (with Pt/C as CE/RE in a humidified atmosphere of H_2_ in Ar), evidencing redox features and an ECSA comparable to those obtained in 5 cm^2^ single-cell PEMFCs. To further investigate the SMSI effect in the Pt/TiO_*x*_/C catalyst, key operating potentials of interest (i.e., within the H_UPD_, the double-layer capacitance, and the Pt-oxide formation regions) were applied and operando NAP-XPS spectra were acquired. As observed from the electrochemical results, Pt/TiO_*x*_/C exhibits less oxidation upon potential increase compared to Pt/C, due to the SMSI-induced TiO_*x*_ layer on the Pt surface. However, the data strongly indicate a residual oxidation of the Pt/TiO_*x*_/C catalyst, likely originating from Pt particles supported on carbon instead of on the TiO_*x*_ particles. This thesis is supported by the fact that also for Pt/TiO_*x*_/C the relative trend of the Pt oxidation with increasing potential follows that of the Pt/C reference catalyst in both electrochemical and operando NAP-XPS data. TEM analysis of the Pt/TiO_*x*_/C catalyst powder reveals the presence of Pt particles deposited on carbon, confirming that the Pt/TiO_*x*_/C catalyst in this study is not a perfectly pure phase. Nevertheless, the reduced oxidation across all potentials compared to Pt/C matches well with the observed ORR mass activity in 5 cm^2^ single-cell PEMFC measurements from the literature, indicating a residual amount of Pt on carbon of ≈33–40%.^9^ Although the catalyst used was a mixed phase, the reduction of Pt-oxide formation in Pt/TiO_*x*_/C to 40% vs Pt/C still strongly suggests an extreme suppression of Pt oxidation at high potentials due to the TiO_*x*_ layer, explaining the high retention of HOR activity at ≥1.0 V_RHE_ and its low activity toward ORR from RDE experiments that were reported in the literature.

Regarding the Pt-oxide quantification, the good quality of the electrochemical data provided by the optimized operando NAP-XPS cell allows us to infer that the discrepancies in the absolute values in the NAP-XPS oxide quantification are likely due to cracks in the BLG film, leading to a dry-out of the near-surface region of the WE (i.e., near the WE/BLG/vacuum interface), thereby partially hindering Pt-oxide formation on the Pt particles in this region due to the lack of water; this occurs only within the XPS detection depth, without actually affecting the bulk of the working electrode and thus the electrochemical data. Nonetheless, examining relative changes between Pt/TiO_*x*_/C and Pt/C in Pt oxidation upon potential increases from both electrochemistry and XPS still reveals the key difference between the two catalysts. Even though a mixed phase of catalyst was used, the results suggest that a vastly different potential-dependent Pt oxidation, i.e., barely any oxidation is expected for fully encapsulated pure Pt/TiO_*x*_/C catalysts.

## Supplementary Material



## Notes

Electrochemical and operando NAP-XPS data with and without bilayer graphene; estimation of the relative humidity in the operando cell by comparison with PEMFC measurements; contribution of the Ti 3s plasmon to the Pt 4f region in the operando samples; estimation of the information depth in the operando NAP-XPS samples; overview of XPS fitting parameters (PDF)

## ∇

Alba Synchrotron Light Source, Experiments Division, Cerdanyola del Vallés, Barcelona 08290, Spain

## ○

Bosch Research and Technology Center North America, Robert Bosch LLC, 384 Santa Trinita Ave, Sunnyvale, CA 94085, United States
