# Supplementary material for: Elucidating the Hydrogen Selectivity of Pt/TiO x /C as a Fuel-Cell Catalyst by Operando Near-Ambient-Pressure XPS
Source: ACS Appl Mater Interfaces. 2025 Aug 30;17(36):50626–38. doi: 10.1021/acsami.5c09047 (PMC12442003; doi:10.1021/acsami.5c09047)
Supplement: Supplementary file 1 [file am5c09047_si_001.pdf]

Supporting Information

Elucidating the Hydrogen Selectivity of Pt/TiO_x/C as a Fuel-Cell Catalyst by Operando Near-Ambient-Pressure XPS

Nhat Long Tran Pham[1,z,=], Simon Qian[1,z,=], Thomas Götsch[2], Corbinian Grön[1], Juan Jesus Velasco-Vélez[2,3,], Björn M. Stühmeier[1,§], Axel Knop-Gericke[2,3], Hubert A. Gasteiger[1] & Michele Piana[1]*

[1] Technical University of Munich, TUM School of Natural Sciences, Department of Chemistry and Catalysis Research Center, Chair of Technical Electrochemistry, Lichtenbergstr. 4, 85748 Garching b. München, Germany

[2] Fritz-Haber-Institut der Max-Planck-Gesellschaft, Department of Inorganic Chemistry, Faradayweg 4-6, 14195 Berlin, Germany

[3] Max Planck Institute for Chemical Energy Conversion, Stiftstraße 34-36, Mülheim an der Ruhr 45470, Germany

[*] Current address: Alba Synchrotron Light Source, Experiments Division, Cerdanyola del Vallés, Barcelona 08290, Spain

[§] Current address: Bosch Research and Technology Center North America, Robert Bosch LLC, 384 Santa Trinita Ave, Sunnyvale, CA 94085, United States

[z] Corresponding Authors.
E-mail Address [nhat-long.tran-pham@tum.de]
E-mail Address [simon.qian@tum.de]

[=] These authors contributed equally to this work.

S1. Electrochemical and operando NAP-XPS data with and without BLG

To validate the influence of the bilayer graphene (BLG) film deposited on the working electrode, an operando measurement without BLG was performed. In terms of electrochemistry, as shown in Figure S1, all features are identical, with minor variations in absolute current, mainly due to sample variation. These results show that the BLG is not expected to affect the general electrochemistry of the ≈ 6-8 μm thick standard PEMFC catalyst layer. Therefore, a similar average relative humidity (RH) within the WE is expected in both cases since there is no apparent major shift in overpotential of the Pt-oxide feature.

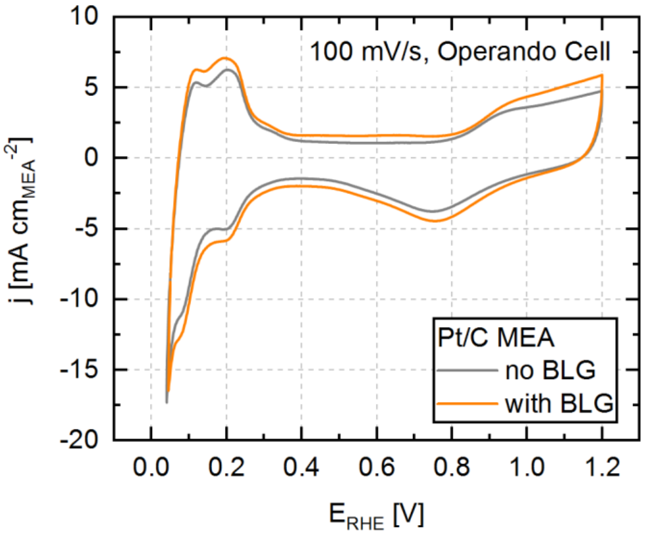

Figure S1. CVs collected on MEAs with Pt/C as WE with a loading of ≈ 45 ± 10 $\mu g_{Pt}\, cm_{MEA}^{-2}$ and an I/C (ionomer/carbon ratio) of ≈ 0.65, using the operando cell at the ISISS/BESSY II (see Figure 1, main text) at RT, with a scan rate of 100 mV s^{-1}, between 0.045-1.2 V_{RHE}, and applying a dynamic vacuum of ≈ 0.02 mbar in the WE compartment. The operando cell was built with BLG (solid orange line) and without BLG (solid grey line).

In Figure S2, operando NAP-XPS spectra are shown for an MEA with and without BLG at 0.15 V_{RHE} (H_{UPD} region) and 1.4 V_{RHE} (Pt-oxide region). As Mom et al. already showed, the BLG acts as an evaporation barrier to keep the XPS-probed surface of the catalyst layer humid and, therefore, to allow Pt oxidation at the expected high potentials (Figure S2b).[1]

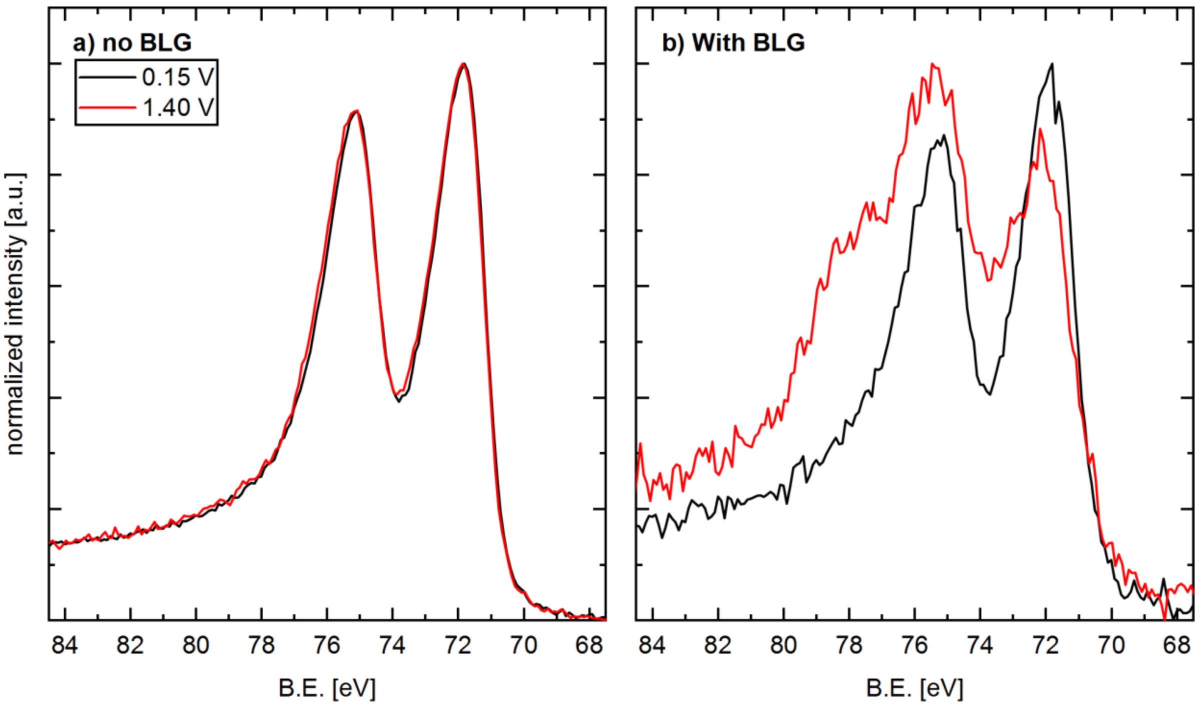

Figure S2. C 1s energy-calibrated NAP-XPS spectra of the Pt 4f region (86-66 eV_{bin}) using an MEA with Pt/C as WE, measured at an excitation energy of 871 eV, dynamic vacuum of ≈ 0.02 mbar, 10 scans, 0.1 s_{dwell}, 0.1 eV/step at 0.15 V (solid black line) and 1.40 V (solid red line). (**a**)**:** MEA without BLG and (**b**)**:** with BLG.

Without BLG, as shown in Figure S2a, Pt shows no oxidation, even though the electrochemistry from the bulk of the electrode clearly indicates the formation of Pt oxide at ≥ 1.00 V_{RHE} in Figure S1; as a consequence, no humidification must be present within the first few nm of the catalyst layer. This result shows the difference between probing the whole electrode (≈ 6-8 µm) in the electrochemical data and probing only a few nm within the XPS detection depth.

S2. Relative humidity estimation by comparison with PEMFC measurements

A 5 cm² single-cell proton exchange membrane fuel cell (PEMFC) was built using an MEA with Pt/C as WE, as described in the experimental section of the main text. Details of the used fuel cell equipment and hardware are described elsewhere.[2] Prior to any electrochemical measurements, a conditioning cycle was applied at 80°C, 150 kPa_{abs}, 100% RH, H_2/air = 1394/3323 nccm, by running 10 cycles of 0.60 V_{RHE} for 45 minutes, 0.95 V_{RHE} for 5 minutes and 0.85 V_{RHE} for 10 minutes to activate and humidify the MEA and clean the catalyst from impurities. To replicate the humidification in the operando cell as closely as possible, the RH of the WE was kept to RH_{WE} =0%, whereas various RHs for the reference/counter electrode (CE/RE) in the range of 0-70% RH were set. Each RH condition was then equilibrated at 40 °C, ambient pressure and a flow of N_2/N_2 =1000/1000 nccm (CE/WE) for one hour. Before the CVs were measured, the gases were switched to 5% H_2 in Ar/N_2 = 200/50 nccm (CE/WE) for 10 minutes, followed by completely cutting off the WE nitrogen flow to zero. The CVs and equilibration of the RHs were measured from low to high RH at 100 mV s^{-1} between 0.07 and 1.00 V_{RHE}. A modest temperature of 40°C was chosen as the lowest possible in the fuel cell test station but low enough to be able to fairly compare the CVs in PEMFCs with those collected using the operando cell. Figure S3 shows the various CVs under different RHs compared to the operando cell measurement, for which the current was scaled due to its higher double layer capacitive current at 0.45 V_{RHE} (≈ 1.7 mA cm^{-2}) to align with the PEMFC measurement (≈ 1.0 mA cm^{-2}). This may arise from the different setup and conditioning performed. As the primary indicator for RH, the overpotential of the Pt-oxide feature from ≥ 0.7 V_{RHE} was used, since the Pt oxidation itself is rather sensitive to RH changes, leading to a higher overpotential with decreasing RH.[3,4] As the operando cell (orange CV) has a possible RH between 0% and 40%, it can be concluded that the operando MEA is not completely

dried out, but also does not reach a modest RH of 40%. Since the current is different from zero, a certain degree of humidification reaches the working electrode while collecting operando data, which is essential to allow for the oxidation of Pt.

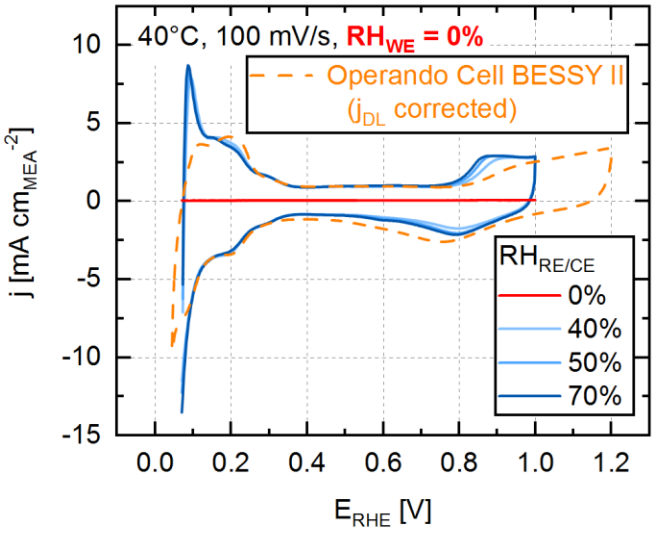

Figure S3. Cyclic voltammograms at various RHs in a 5 cm² single-cell PEMFC (red and blue solid lines) compared to the CV collected at BESSY II with the operando cell (orange dashed line), all collected using MEAs with Pt/C as WE. Before the CVs were recorded, the 5 cm² single-cell PEMFC was conditioned at 80°C, 150 kPa_{abs}, 100% RH, H_2/air = 1394/3323 nccm and 10 cycles of 0.60 (45 minutes) → 0.95 (5 minutes) → 0.85 V_{RHE} (10 minutes). Each RH condition was held for 1 hour at 40 °C, RH_{WE} = 0%, ambient pressure, and varying $RH_{CE/RE}$. CVs were recorded with a scan rate of 100 mV s^{-1} between 0.07 and 1.0 V_{RHE}. The current in the CV of the operando cell was scaled to the double layer current j_{DL} of the PEMFC to better compare the results.

S3. LA line shape and contribution of the Ti 3s plasmon to the Pt 4f region

The fitting parameters of the Lorentzian Asymmetric (LA) line shape were determined using CasaXPS (Version 2.3.25PR1.0), which includes the B. E. positions of the Pt $4f_{7/2}$ feature, their FWHM, and their atomic percentages based on the integrals for the fitting as shown in Figure 3 of the main text. A short description of the fitting parameters for the line shape function is provided by the notation:[5–7]

$$LA\ (\alpha, \beta, \omega)$$

where the parameters α and β affect the slope of the Lorentzian tail of the line shape for higher and lower binding energies, respectively, with a higher value resulting in a steeper edge of the respective tail. Asymmetry is induced by introducing $\alpha \neq \beta$. The parameter ω is related to the contribution of the width of the Gaussian function, which is convoluted into the Lorentzian function. Accordingly, the asymmetric line shapes LA(1.2,50,100) for Pt $4f_{7/2}$ and LA(1.2,50,40) for Pt $4f_{5/2}$ were used for the $Pt^{0,\ \delta+}$ species due to its metallic nature. This changed to a less asymmetrical line shape of LA(1.5,2.2,0) of both the Pt $4f_{7/2}$ and Pt $4f_{5/2}$ features for the Pt^{4+} oxidation state detected in PtO_2. All resulting B. E. positions listed in Table 1 of the main text are well in agreement with the literature. In the case of PtO_2, $Pt^{0,\ \delta+}$ was found, since the powder was used as received. For the sake of simplicity, a differentiation was not made between Pt^0 and $Pt^{\delta+}$ and is defined as $Pt^{0,\ \delta+}$, especially because the Pt in the MEA is surrounded by multiple possible adsorbents such as the ionomer, water, or even the TiO_x overlayer in the case of Pt/TiO_x/C. A spin-orbit splitting between Pt $4f_{7/2}$ and Pt $4f_{5/2}$ of 3.34 eV was applied, with the sole exception in the spin-orbit splitting of 3.75 eV for $Pt^{0,\ \delta+}$ species of the Pt/TiO_x/C reference sample. As mentioned

in the main text, this was done to accurately reconstruct the raw data from the reference measurement at a potential of 0.15 V_{RHE}.

To assess the areal contribution of a Ti 3s plasmon in the Pt 4f region, reference measurements of a heat-treated TiO_x powder were performed using a laboratory-based XPS (Axis Supra, Kratos, UK). The heat-treatment of the TiO_x sample was performed according to Stühmeier et al. and is necessary to have a fair comparison to the Pt/TiO_x/C catalyst, since the heat-treatment enables the TiO_x to encapsulate the Pt particle. For the XPS data collection, the powder sample was prepared and measured in ultra-high vacuum (UHV) around 10^{-8} mbar with monochromatized Al-K_α radiation at 1486.6 eV. The pass energy was set to 40 eV, with a step size of 0.1 eV and dwell times of 1 s, while charge neutralization was employed. The spectra were energy calibrated to the adventitious carbon feature in the C 1s region and analyzed using the Gaussian-Lorentzian product function GL(30), incorporating 30% Gaussian character, after the subtraction of a Shirley-background. As evident in Figure S4, the Ti 3s plasmon is observed at around 75 eV within the Pt 4f region. However, its contribution is relatively minor, amounting to only roughly 10% of the Ti 3s area and being significantly less when compared to the Ti 2p area (<1%). Since the Ti 2p region was measured at every potential of the operando experiment, the Ti 3s plasmon contribution referenced against the Ti 2p signal is reduced to less than 1% of the total Ti 2p area. Based on this percentage, the expected Ti 3s plasmon area can be calculated using the Ti 2p area from the operando measurements, which is about 1-2% of the measured Pt 4f area. Therefore, any significant areal contribution of the Ti 3s plasmon to the Pt 4f fits can be ruled out. However, as illustrated in Figure S4, the plasmon appears to elevate the background up to a binding energy of approximately 80 eV. This background elevation may contribute to the observed enlarged spin-orbit splitting of the Pt 4f doublet. While this observation is noted, the focus of this work is on the

fitting of the obtained Pt 4f data. Thus, the reasons behind the increased spin-orbit splitting are beyond the scope of this study.

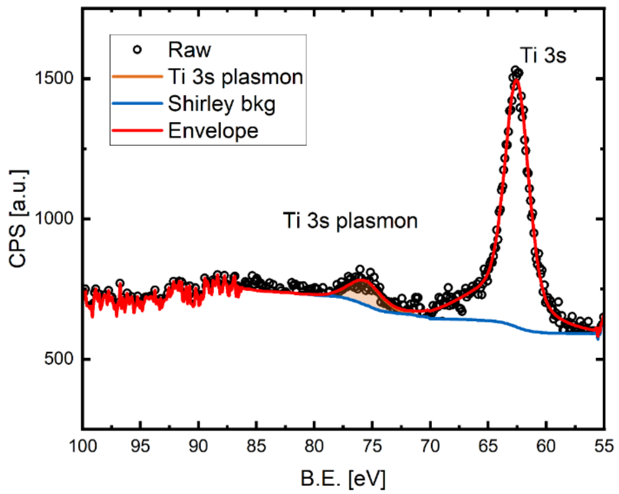

Figure S4. Reference measurement of a heat-treated TiO_x powder in a laboratory-based XPS (Axis Supra, Kratos, UK) to estimate the areal contribution of the Ti 3s plasmon in the Pt 4f region. The Ti 3s region is shown after C 1s energy calibration with its raw data (black hollow dots), the highlighted Ti 3s plasmon (orange), the applied Shirley-background (blue), and the envelope obtained from fitting the entire region of interest with GL(30) functions (red).

S4. Estimation of the information depth for our operando NAP-XPS setup

To estimate the XPS information depth (defined as the thickness from which 95% of the photoelectron signal is detected), effective attenuation lengths (EALs) are obtained using the NIST Electron Effective-Attenuation-Length Database (software version 1.3) for the overlayers (BLG, ionomer, SMSI-induced TiO_x layer for Pt/TiO_x/C) and the Pt particle. The parameters used to obtain each EAL are listed in Table S1.

Table S1. Parameters used to calculate the EALs of each overlayer originate from the NIST Electron Effective-Attenuation-Length Database including the user guide (software version 1.3)[8] for a kinetic energy of 800 eV and 54° incidence angle between X-ray incident beam and sample surface. Rutile TiO_2 was used to estimate the SMSI-induced TiO_x layer, and the thickness was assumed to be twice the atomic radius of Ti (0.147 nm). For the ionomer, the asymmetry parameter β (assuming Al Kα X-rays as an approximation), the band gap, and the density were assumed to be comparable to PTFE, whereas the amount of valence electrons (VE) is based on the molecular formula of Nafion ($C_7HF_{13}O_5S \cdot C_2F_4$). Furthermore, the thickness of the ionomer layer was estimated using a density of 2 g cm^{-3}, a theoretical surface area of 216 m^2 g^{-1} for the Pt/C catalyst (based on the ECSA of the Pt/C catalyst and the BET surface of 245.9 m^2 g^{-1} for Vulcan carbon). Carbon (graphite) parameters were used for the BLG, with a thickness after subtraction of the interlayer space in graphite. I_d/I_0 were calculated using equation (S.1), which is also used for the layer thicknesses of Pt/C and Pt/TiO_x/C at an overall I_d/I_0 of 0.05 (our information depth).

	β	VE	Band gap [eV]	Density [g cm^{-3}]	EAL [nm]	Layer thickness d [nm]	I_d/I_0
TiO_2 (rutile)	1.41	16	3.05	4.24	1.5156	0.294	0.82
Ionomer	1.00	192	5.00	2.00	2.2148	1.200	0.58
BLG (C)	2.00	–	–	–	2.1104	0.355	0.85
Pt	1.04	–	–	21.45	0.7525	1.7	0.10
Pt/C	–	–	–	–	–	1.7	0.05
Pt	1.04	–	–	21.45	0.7550	1.55	0.13
Pt/TiO_x/C	–	–	–	–	–	1.55	0.05

Using the Lambert-Beer law modified for XPS and assuming an emission angle of 0° (see equation (S.*1*), these EALs can be used to estimate the attenuation of the emitted photoelectron (I_d/I_0) at a known thickness d.

$$\frac{I_d}{I_0} = e^{-d/EAL_d} \tag{S.1}$$

Knowing the attenuation of each overlayer, their product is the combined attenuation, leading to an $I_d/I_0 \approx 0.49$ and ≈ 0.40 for Pt/C and Pt/TiO_x/C, respectively. Based on this, the information depths (defined as d at $I_d/I_0 = 0.05$) for Pt/C and Pt/TiO_x/C are equal to 1.7 and 1.55 nm, respectively, as calculated from equation (S.1) and using the EAL for Pt at those thicknesses (see Table S1). Since, for geometrical reasons, the surface-to-bulk ratio of a spherical particle with a diameter of 3 nm stays constant from a depth of 1.3 nm onwards, we can be confident that the at% of the Pt species obtained by the XPS fitting are representative for the entire particle.

S5. Overview of fitting parameters for the operando data set

Here we report the detailed information on the fitting parameters of the components using the synthetic line shapes, based on the LA line shapes from the reference samples as well as on the operando data of the MEAs with Pt/C and Pt/TiO_x/C as WEs.

Table S2. Detailed fitting parameters obtained for the operando data of the MEA with Pt/C as WE using the synthetic line shapes for the different components. The peak position of the Pt $4f_{7/2}$ represents the B.E. value at the peak maximum. Besides the peak position of the Pt $4f_{7/2}$ and the at% of the Pt species, a FWHM scaling factor compared to the reference synthetic line shape is provided as well. It must be noted that this MEA was only measured once.

**Pt/C-MEA**					
**Potential**	**Component**	**Synthetic Line Shape**	**Peak Position Pt $4f_{7/2}$ [eV]**	**FWHM Scaling Factor [-]**	**at%**
**0.15 V_{RHE}**	**$Pt^{0,\delta+}$**	$Pt^{0,\delta+}_{Pt/C}$ at 0.15 V_{RHE}	72.00	1.01	100
**0.45 V_{RHE}**	**$Pt^{0,\delta+}$**	$Pt^{0,\delta+}_{Pt/C}$ at 0.15 V_{RHE}	72.04	1.01	100
**1.0 V_{RHE}**	**$Pt^{0,\delta+}$**	$Pt^{0,\delta+}_{Pt/C}$ at 0.15 V_{RHE}	72.12	1.05	92.68
	**Pt^{4+}**	$Pt^{4+}_{PtO_2\ Powder}$ ex situ	75.01	1.23	7.32
**1.2 V_{RHE}**	**$Pt^{0,\delta+}$**	$Pt^{0,\delta+}_{Pt/C}$ at 0.15 V_{RHE}	72.17	0.99	87.74
	**Pt^{2+}**	$Pt^{4+}_{PtO_2\ Powder}$ ex situ	74.00	0.85	6.14
	**Pt^{4+}**	$Pt^{4+}_{PtO_2\ Powder}$ ex situ	75.50	0.96	6.12
**1.4 V_{RHE}**	**$Pt^{0,\delta+}$**	$Pt^{0,\delta+}_{Pt/C}$ at 0.15 V_{RHE}	72.24	1.15	69.88
	**Pt^{2+}**	$Pt^{4+}_{PtO_2\ Powder}$ ex situ	74.50	0.96	10.37
	**Pt^{4+}**	$Pt^{4+}_{PtO_2\ Powder}$ ex situ	75.05	1.12	19.75
**1.4 V_{RHE} (EoT)**	**$Pt^{0,\delta+}$**	$Pt^{0,\delta+}_{Pt/C}$ at 0.15 V_{RHE}	72.35	1.08	62.48
	**Pt^{2+}**	$Pt^{4+}_{PtO_2\ Powder}$ ex situ	74.35	0.96	15.90
	**Pt^{4+}**	$Pt^{4+}_{PtO_2\ Powder}$ ex situ	75.23	1.05	21.62

Table S3. Detailed fitting parameters obtained from the operando data on the MEA with $Pt/TiO_x/C$ as WE using the synthetic line shapes for the different Pt oxidation states. Average data and errors are calculated from the two measured MEAs in the operando cell used as minimum and maximum. The peak position of the Pt $4f_{7/2}$ represents the B.E. value at the peak maximum. Besides the peak position of the Pt $4f_{7/2}$ and the at% of the Pt species, a FWHM scaling factor compared to the reference synthetic line shape is provided as well.

$Pt/TiO_x/C$-MEA

Potential	Component	Synthetic Line shape	Peak Position Pt $4f_{7/2}$ [eV]	FWHM Scaling Factor [-]	at%
**0.15 V_{RHE}**	**$Pt^{0, \delta+}$**	$Pt^{0, \delta+}_{Pt/TiO_x/C}$ at 0.15 V_{RHE}	72.10 ± 0.04	1.01 ± 0.01	100
**0.45 V_{RHE}**	**$Pt^{0, \delta+}$**	$Pt^{0, \delta+}_{Pt/TiO_x/C}$ at 0.15 V_{RHE}	72.06 ± 0.04	1.01 ± 0.02	100
**1.0 V_{RHE}**	**$Pt^{0, \delta+}$**	$Pt^{0, \delta+}_{Pt/TiO_x/C}$ at 0.15 V_{RHE}	72.08 ± 0.02	0.99 ± 0.01	98 ± 2
	**Pt^{4+}**	$Pt^{4+}_{PtO_2\ Powder}$ ex-situ	75.30	1.1	2 ± 2
**1.2 V_{RHE}**	**$Pt^{0, \delta+}$**	$Pt^{0, \delta+}_{Pt/TiO_x/C}$ at 0.15 V_{RHE}	72.06 ± 0.01	1.04 ± 0.02	92 ± 1
	**Pt^{2+}**	$Pt^{4+}_{PtO_2\ Powder}$ ex situ	73.9 ± 0.4	1.36 ± 0.04	3 ± 1
	**Pt^{4+}**	$Pt^{4+}_{PtO_2\ Powder}$ ex situ	74.9 ± 0.2	1.28 ± 0.01	4.26 ± 0.07
**1.4 V_{RHE}**	**$Pt^{0, \delta+}$**	$Pt^{0, \delta+}_{Pt/TiO_x/C}$ at 0.15 V_{RHE}	72.1 ± 0.11	1.01 ± 0.01	84 ± 2
	**Pt^{2+}**	$Pt^{4+}_{PtO_2\ Powder}$ ex situ	74.0 ± 0.5	1.02 ± 0.08	4.9 ± 0.12
	**Pt^{4+}**	$Pt^{4+}_{PtO_2\ Powder}$ ex situ	74.96 ± 0.06	1.19 ± 0.10	11 ± 2
**1.4 V_{RHE} (EoT)**	**$Pt^{0, \delta+}$**	$Pt^{0, \delta+}_{Pt/TiO_x/C}$ at 0.15 V_{RHE}	72.04	0.98	81.39
	**Pt^{2+}**	$Pt^{4+}_{PtO_2\ Powder}$ ex situ	73.77	0.93	6.40
	**Pt^{4+}**	$Pt^{4+}_{PtO_2\ Powder}$ ex situ	75.24	1.01	12.21

The MEA with Pt/C as WE was only measured once. The statistical error is expected to be similar to the one obtained from the MEAs with $Pt/TiO_x/C$ as WE, reported in. Furthermore, the presence of Pt^{4+} was found only for one $Pt/TiO_x/C$ measurement at 1.0 V_{RHE}, suggesting a low degree of oxidation. Error bars at 1.4 V_{RHE} (end of test, EoT) for the $Pt/TiO_x/C$ are missing, since this measurement point was only measured once due to time constraints. Furthermore, the FWHM scaling factor describes the broadening or compression of the used synthetic line shape function.

Table S4 shows the average binding energies of the Pt $4f_{7/2}$ feature and their FWHM scaling factors for all the Pt oxidation states, resulting in high reproducibility and minimal deviation across all investigated potentials, especially considering the variation in samples, spots and potentials.

Table S4. Average binding energies at the peak maximum of each Pt species for Pt $4f_{7/2}$ and their FWHM scaling factors from the operando data sets of all MEAs with Pt/C and Pt/TiO_x/C (two repeated measurements) as WEs at all potentials based on synthetic line shapes. The errors are the standard deviations of the population calculated based on the entire data sets.

	**Component**	**Synthetic Line shape**	**Peak Position Pt $4f_{7/2}$ [eV]**	**FWHM scaling factor [-]**
**Pt/C (across all potentials)**	**$Pt^{0,\delta+}$**	$Pt^{0,\delta+}_{Pt/TiO_x/C}$ at 0.15 V_{RHE}	72.1 ± 0.12	1.05 ± 0.05
	**Pt^{2+}**	$Pt^{4+}_{PtO_2\ Powder}$ ex situ	74.3 ± 0.2	0.92 ± 0.05
	**Pt^{4+}**	$Pt^{4+}_{PtO_2\ Powder}$ ex situ	75.2 ± 0.2	1.09 ± 0.10
**Pt/TiO_x/C (across all potentials)**	**$Pt^{0,\delta+}$**	$Pt^{0,\delta+}_{Pt/TiO_x/C}$ at 0.15 V_{RHE}	72.07 ± 0.06	1.05 ± 0.05
	**Pt^{2+}**	$Pt^{4+}_{PtO_2\ Powder}$ ex situ	73.9 ± 0.4	0.92 ± 0.09
	**Pt^{4+}**	$Pt^{4+}_{PtO_2\ Powder}$ ex situ	75.1 ± 0.2	1.07 ± 0.09

References

(1) Mom, R.; Frevel, L.; Velasco-Vélez, J. J.; Plodinec, M.; Knop-Gericke, A.; Schlögl, R. The Oxidation of Platinum under Wet Conditions Observed by Electrochemical X-Ray Photoelectron Spectroscopy. *J. Am. Chem. Soc.* **2019**, *141* (16), 6537–6544. https://doi.org/10.1021/jacs.8b12284.

(2) Stühmeier, B. M.; Damjanović, A. M.; Rodewald, K.; Gasteiger, H. A. Selective Anode Catalyst for the Mitigation of Start-up/Shut-down Induced Cathode Degradation in Proton Exchange Membrane Fuel Cells. *J. Power Sources* **2023**, *558* (December 2022), 232572. https://doi.org/10.1016/j.jpowsour.2022.232572.

(3) Xu, H.; Kunz, R.; Fenton, J. M. Investigation of Platinum Oxidation in PEM Fuel Cells at Various Relative Humidities. *Electrochem. Solid-State Lett.* **2007**, *10* (1), 1–6. https://doi.org/10.1149/1.2372230.

(4) Liu, Y.; Murphy, M. W.; Baker, D. R.; Gu, W.; Ji, C.; Jorne, J.; Gasteiger, H. A. Proton Conduction and Oxygen Reduction Kinetics in PEM Fuel Cell Cathodes: Effects of Ionomer-to-Carbon Ratio and Relative Humidity. *J. Electrochem. Soc.* **2009**, *156* (8), B970. https://doi.org/10.1149/1.3143965.

(5) Major, G. H.; Avval, T. G.; Patel, D. I.; Shah, D.; Roychowdhury, T.; Barlow, A. J.; Pigram, P. J.; Greiner, M.; Fernandez, V.; Herrera-Gomez, A.; Linford, M. R. A Discussion of Approaches for Fitting Asymmetric Signals in X-ray Photoelectron Spectroscopy (XPS), Noting the Importance of Voigt-like Peak Shapes. *Surf. Interface Anal.* **2021**, *53* (8), 689–707. https://doi.org/10.1002/sia.6958.

(6) Walton, J.; Wincott, P.; Fairley, N.; Carrick, A. Peak Fitting with CasaXPS. *Casa XPS* **2010**, 1–140.

(7) Biesinger, M. C.; Payne, B. P.; Grosvenor, A. P.; Lau, L. W. M.; Gerson, A. R.; Smart, R. S. C. Resolving Surface Chemical States in XPS Analysis of First Row Transition Metals, Oxides and Hydroxides: Cr, Mn, Fe, Co and Ni. *Appl. Surf. Sci.* **2011**, *257* (7), 2717–2730. https://doi.org/10.1016/j.apsusc.2010.10.051.

(8) Powell, C. J.; Jablonski, A. *NIST Electron Effective-Attenuation-Length Database - Version 1.3*; National Institute of Standards and Technology: Gaithersburg, MD, 2011. https://doi.org/10.1384/jsa.9.322.
